# Health system costs for individual and comorbid noncommunicable diseases: An analysis of publicly funded health events from New Zealand

**DOI:** 10.1371/journal.pmed.1002716

**Published:** 2019-01-08

**Authors:** Tony Blakely, Giorgi Kvizhinadze, June Atkinson, Joseph Dieleman, Philip Clarke

**Affiliations:** 1 Burden of Disease Epidemiology, Equity and Cost-Effectiveness Programme, Department of Public Health, University of Otago, Wellington, New Zealand; 2 Melbourne School of Population and Global Health, University of Melbourne, Melbourne, Australia; 3 Institute of Health Metrics and Evaluation, University of Washington, Seattle, Washington, United States of America; 4 Health Economics Research Centre, Nuffield Department of Population Health, University of Oxford, Oxford, United Kingdom; Edinburgh University, UNITED KINGDOM

## Abstract

**Background:**

There is little systematic assessment of how total health expenditure is distributed across diseases and comorbidities. The objective of this study was to use statistical methods to disaggregate all publicly funded health expenditure by disease and comorbidities in order to answer three research questions: (1) What is health expenditure by disease phase for noncommunicable diseases (NCDs) in New Zealand? (2) Is the cost of having two NCDs more or less than that expected given the independent costs of each NCD? (3) How is total health spending disaggregated by NCDs across age and by sex?

**Methods and findings:**

We used linked data for all adult New Zealanders for publicly funded events, including hospitalisation, outpatient, pharmaceutical, laboratory testing, and primary care from 1 July 2007 to 30 June 2014. These data include 18.9 million person-years and $26.4 billion in spending (US$ 2016). We used case definition algorithms to identify if a person had any of six NCDs (cancer, cardiovascular disease [CVD], diabetes, musculoskeletal, neurological, and a chronic lung/liver/kidney [LLK] disease). Indicator variables were used to identify the presence of any of the 15 possible comorbidity pairings of these six NCDs. Regression was used to estimate excess annual health expenditure per person. Cause deletion methods were used to estimate total population expenditure by disease. A majority (59%) of health expenditure was attributable to NCDs. Expenditure due to diseases was generally highest in the year of diagnosis and year of death. A person having two diseases simultaneously generally had greater health expenditure than the expected sum of having the diseases separately, for all 15 comorbidity pairs except the CVD-cancer pair. For example, a 60–64-year-old female with none of the six NCDs had $633 per annum expenditure. If she had both CVD and chronic LLK, additional expenditure for CVD separately was $6,443/$839/$9,225 for the first year of diagnosis/prevalent years/last year of life if dying of CVD; additional expenditure for chronic LLK separately was $6,443/$1,291/$9,051; and the additional comorbidity expenditure of having both CVD and LLK was $2,456 (95% confidence interval [CI] $2,238–$2,674). The pattern was similar for males (e.g., additional comorbidity expenditure for a 60–64-year-old male with CVD and chronic LLK was $2,498 [95% CI $2,264–$2,632]). In addition to this, the excess comorbidity costs for a person with two diseases was greater at younger ages, e.g., excess expenditure for 45–49-year-old males with CVD and chronic LLK was 10 times higher than for 75–79-year-old males and six times higher for females. At the population level, 23.8% of total health expenditure was attributable to higher costs of having one of the 15 comorbidity pairs over and above the six NCDs separately; of the remaining expenditure, CVD accounted for 18.7%, followed by musculoskeletal (16.2%), neurological (14.4%), cancer (14.1%), chronic LLK disease (7.4%), and diabetes (5.5%). Major limitations included incomplete linkage to all costed events (although these were largely non-NCD events) and missing private expenditure.

**Conclusions:**

The costs of having two NCDs simultaneously is typically superadditive, and more so for younger adults. Neurological and musculoskeletal diseases contributed the largest health system costs, in accord with burden of disease studies finding that they contribute large morbidity. Just as burden of disease methodology has advanced the understanding of disease burden, there is a need to create disease-based costing studies that facilitate the disaggregation of health budgets at a national level.

## Abstract 2

Tony Blakely and colleagues use national health data for New Zealand to estimate the costs of individual diseases as well as whether multiple morbidities add an additional cost above the sum of the expected costs of the individual diseases.

## Author summary

**Why was this study done?:**

- Few studies have estimated disease-specific health system expenditure for many diseases simultaneously, keeping within the total envelope of health expenditure. Most cost of illness studies focus on a single or small set of disease(s) and overestimate costs due to attribution of comorbidities.
- Few studies have estimated whether having two or more diseases increases or decreases health system expenditure, over and above that expected from having the diseases separately.

**What did the researchers do and find?:**

- Using nationally linked health data for all New Zealanders, the additional or excess costs from having one or more chronic diseases were calculated using regression equations.
- Nearly a quarter of all health expenditure (23.8%) on chronic noncommunicable disease was attributable to costs from having two or more diseases (i.e., costs due to comorbid conditions, over and above separate costs from single diseases).
- Of the remaining three quarters of health expenditure, its attribution across six chronic diseases (as though they were the only disease a person had) were: heart disease and stroke, 18.7%; musculoskeletal, 16.2%; neurological, 14.4%; cancer, 14.1%; chronic lung, liver, or kidney disease, 7.4%; and diabetes, 5.5%.

**What do these findings mean?:**

- Health system expenditure due to musculoskeletal and neurological diseases is sizeable, suggesting these diseases need more policy and planning consideration and research than they currently receive.
- The additional costs from having two or more diseases, over and above the costs of having disease separately, reinforce the need for policy and planning to anticipate the effects of aging populations with comorbidity.

## Introduction

The Global Burden of Disease (GBD) has achieved the vision of a coherent and comparable set of epidemiological estimates of disease-specific incidence, prevalence, morbidity, and mortality across all countries and since 1990 [1,2]. There are many reasons associated with planning and comparative analysis that suggest it would also be worthwhile to pursue a similar vision of national health system expenditure, disaggregating spending by disease in a manner comparable across countries. The Organization of Economically Developed Countries has promulgated such a vision under the rubric of national health system accounts that disaggregates total health expenditure by disease [3], and within the United States, there have been proposals for the creation of such accounts [4]. A comparable set of disease costs across multiple countries would facilitate comparisons both over time within countries and between countries. Such accounts would provide measures of disease burden that would generate a consistent set of costs to use in cost of illness (COI) and cost-effectiveness studies. Achieving such a vision, though, is challenging. Countries vary in the structuring of their health systems, and there is a need for standardisation of methods across countries. Using the same disease classification systems and standardising, or at least accommodating differing boundaries of total health expenditure, is critical [5].

A key limitation of disease-specific COI studies that focus on a single or small set of disease(s) is their tendency to overestimate costs due to attribution of comorbidities. If separate COI studies are naively summed, overestimation of total expenditure is likely [4]. COI studies including most/all diseases in a given country, constraining the total cost to the actual total envelope of health expenditure, are protected against this double-counting problem. Whilst such multiple or all-diseases COI studies are uncommon, the US has a reasonably long history of such studies since the 1960s (e.g., [6] through to more recent studies [7,8]), and examples are emerging in other countries (e.g., [5,9,10]). These studies are usually hybrids of top-down disaggregation and bottom-up estimation using a range of individual-level data sets. However, we are not aware of any studies in which individuals are uniquely identified across multiple data sets (e.g. inpatient, outpatient, pharmaceuticals, mortality, cancer registrations), allowing identification of disease diagnosis, progression, and death (and how costs are aligned to disease progression) and the identification of comorbidities within the same individual, linked to all event and cost data.

Rosen and Cutler (2009) [4,11] outline three approaches to attributing costs to diseases: (1) encounter based, (2) episode based, and (3) person based. Encounter based and episode based both require each encounter or episode to be coded to a primary disease, with associated or comorbid diseases as secondary codes. This coding is routinely undertaken for hospitalisation in many countries but is unlikely to be available for primary care encounters and pharmaceuticals (as many drugs are used for more than one disease). ‘Most cost of illness studies take an encounter-based approach, assigning claims to disease buckets based upon their coded diagnoses. Comorbidities are a major problem here; attributing each spending item for a patient who is both hypertensive and diabetic is not easy’ (p. S10 [4]). Dieleman et al. (2017) have proposed an extension to the encounter-based approach, merged with an attributable risk approach, to determine base costs for each disease and outflows and inflows from comorbid conditions [12]. However, this approach still requires primary and secondary diagnoses for each event. In the person-based approach, all of a person’s total expenditure is regressed on disease indicators and covariates: a model with no disease comorbidity interaction terms will estimate the independent and unconfounded costs of each modelled disease; a model with additional covariates for comorbidity interactions will estimate the greater (or lesser) costs of having two or more diseases simultaneously, over and above that expected for having each disease separately.

In this study, we capitalise on national-level data tracking all publicly funded noncommunicable disease (NCD) health events of uniquely identified individuals, over multiple years, in New Zealand to estimate costs by disease. New Zealand is a high-income country with a burden of NCDs similar to other high-income countries [2], although with less than average healthcare spending per capita than other high-income countries (US$3,648 in purchasing power parity–adjusted US dollars for 2015, compared with a population-weighted average of $5,551 across all high-income countries) [13]. We extend previous work in two ways. First, we attribute health expenditure (1) to mutually exclusive disease categories and (2) split into the single-disease costs (i.e., the cost of disease as though comorbidities did not exist), and the disease comorbidity interactions to capture the impact of people having more than one disease simultaneously.

The second extension is the estimation of costs by phase of disease progression. This includes estimating the costs in year of diagnosis and year of death if dying of that disease. These estimates are useful for subsequent simulation modelling of disease prevention and treatment in cost-effectiveness research, as the differential timing of costs can be better modelled. Our advances over previous national-level and multiple-disease COI studies may not be replicable in many other countries due to data limitations, but we aim to generate a greater depth of understanding to assist not only policy within New Zealand but also the generation of approaches for multicountry COI studies.

We address three specific research questions:

1. What is health expenditure by disease phase for NCDs in New Zealand?
2. Is the cost of having two NCDs more or less than that expected given the independent costs of each NCD?
3. How is total health spending disaggregated by NCDs across age and by sex?

## Materials and methods

### Data

Publicly funded health events make up 82% of all health expenditure in New Zealand, and all such events are assigned a unique personal health identifier, enabling events to be linked across data sets and time by individual. These data have been assigned unit costs since 2007; in this study we used data spanning financial years 2007/2008 to 2013/2014 (i.e., 1 July 2007 to 30 June 2014) for person-year observations among ≥25-year-olds to model costs, but we also use data from previous years to determine if someone has prevalent disease(s) entering the study period. The following national data sets were included: the National Minimum Data Set (NMDS) for all inpatient events since 1990, the nonadmitted patient data set since 1998 (outpatients), cancer registrations since 1995, retail pharmaceuticals since 2005, laboratory claims since 2003, and general medical services claims since May 2000. Retail pharmaceuticals exclude spending in the inpatient facility and are based on the fee schedule, although the government may pay less due to price negotiations. For 97%–98% of the population, we assigned the primary care cost as given by the capitation bulk funding formula based on sex, age, ethnicity, deprivation, and high-user status, while the remaining 2%–3% of the population were not registered with a primary care provider and paid ‘fee for service’ transactions, which are captured in the data. For the included data sets, Ministry of Health cost weights were assigned to each event [14], adjusted for inflation to 2016 real dollars, and then converted to US dollars using the 2016 benchmark purchasing power parity of 1.450 from the Organization for Economic Cooperation and Development (http://stats.oecd.org/Index.aspx?DataSetCode=MEI_PRICES, accessed 20 November 2017).

Data cleaning undertaken by the authors included ensuring same sex, date of birth, and date of death; when an individual had multiple records and there was a disagreement, we used data from the most authoritative source (e.g., mortality record if date of death) or most commonly given (e.g., female sex if on all but one record for a given individual).

### Disease groupings and case definition

For this study, we used two distinct disease groupings. The first set, the aggregated disease set, included the first five of eight disease groupings in the 1990–2013 New Zealand Burden of Disease Study (NZBDS) [15], namely cardiovascular disease (CVD) and diabetes (which we divided into CVD and diabetes separately), cancer, chronic lung/liver/kidney (LLK) disease, neuropsychiatric conditions, and musculoskeletal conditions. The coding and capture of mental events was not yet reliable in the study window, so we excluded psychiatric conditions and had a neurological-only category. The three NZBDS groupings we did not model were as follows: other NCDs; maternal, neonatal, nutritional deficiency, and infectious disorders; and injury. The second set of diseases used in this study were 13 disaggregated diseases, including lung, breast, prostate, colorectal, and other cancers; ischaemic heart disease (IHD), stroke, and other CVD; and chronic lung, chronic liver, and chronic kidney disease.

To determine if any of these diseases were prevalent before or were incident during the 1 July 2007 to 30 June 2014 costing window, a thorough case-finding algorithm was applied, consistent with that used for the NZBDS (**S1 Table**). International Classification of Disease (ICD) codes (primary and other) for events and disease-specific drug and laboratory testing combinations were developed, disease by disease. For cancers, survival after diagnosis by 5 years for lung, 8 years for colorectal, 10 years for ‘other’ cancers, and 20 years for breast and prostate resulted in that person being recoded as free of that cancer, based on statistical cure times [16]. For all other disease, no remission was allowed (i.e., diagnosis was for the remainder of his or her life). Each disease was coded by phase as not present (reference category), diagnosed in that financial year, died in that financial year of that disease, and otherwise prevalent. Note, therefore, the costs for the first two categories are for people with an average of 6 months in that state (but, for the diagnosis category, still including the time and costs for events preceding the diagnosis date in the same financial year).

### Modelling

We did not attempt to classify what healthcare spending was or was not related to each specific disease. Rather, we used the established ‘excess’ or ‘net’ cost approach [17–20], using a statistical approach based on regression models, with total health system cost for each individual in each financial year as the dependent variable and demographics, dummy variables for calendar year, and disease and disease phase indicators as the independent variables. The coefficients for the disease indicators are therefore estimates of the excess cost of having that disease phase and are independent of other diseases (and comorbidities) in the model. The intercept term is the health system cost not attributed to the diseases in the model (e.g., due to injuries, preventive care, mental illness, maternity, etc.).

To model nonadditive costs associated with comorbidity, we interacted the disease indicators in order to identify when an individual had both diseases. It was not possible to include all possible combinations of disease interactions in regression models due to the large number of interaction terms. Thus, we retained for regression modelling the disease phase indicators for each disease as ‘main effects’ but only included all 15 comorbidity pair interactions formed by six diseases (i.e., 6!/([6 − 2]! × 2!) = 15). For example, the cancer–CVD pair was coded ‘1’ for a person-year observation for which the person had a diagnosis of both diseases, regardless of phase of diagnosis (i.e., prevalent cancer with prevalent CVD was coded the same as first year of diagnosis of cancer with prevalent CVD). In regression modelling, the disease phase indicators and disease comorbidity pairs were all interacted with age and age squared, first centring age at the midpoint of the 60–64-year-old age group, 62.5 years, and dividing by 10, so the age interaction terms are interpretable in per 10-year units. Regression equations are detailed in **S1 Appendix**.

To prepare the data for regression modelling, we aggregated them into unique strata formed by cross-classifying 5-year age groups by financial year, by the four levels of each disease (no diagnosis, diagnosed in that financial year, dying of that disease in that financial year, otherwise prevalent with that disease). For the disaggregated 13 disease classifications, there were 151,913 unique strata of these cross-classified categorical variables with at least one male observation, and 139,717 unique strata for females. Between-person ordinary least squares (OLS) regressions were run on these aggregated data sets, with weights equal to the number of observations in each stratum. The advantages of this approach included (1) aggregation reducing the skewness of data, (2) OLS coefficients were directly interpretable as excess costs, (3) the total predicted expenditure (when applying coefficients back onto observations and summing regression-predicted costs across all individuals) always gave the exact observed total expenditure in the data sets, and (4) model-run time was considerably reduced compared with analyses on unit-level data. We also trialled gamma regression on unit-level data, which had better residual plots than OLS but overpredicted disease and total costs, causing us to prefer the above OLS on aggregate data approach. Both because zero costs were so rare (0.09% of all person-year observations) and previous work has found that for estimating averages, a one-part model performs (nearly) as well as a two-part regression [21], we did not employ a two-step process of first estimating individuals with zero expenditure.

Four sets of models were run: disease progression models (models with all the disease-specific phases) for either the aggregated (*n* = 6) or disaggregated (*n* = 13) disease groupings, but without comorbidity interactions; and both these models with the additional 15 disease comorbidity pairs. Note that we retain the same 15 disease comorbidity pairs in the disaggregated 13-disease model, as it was impractical to model and interpret the 78 possible comorbidity pairs formed by 13 diseases.

As part of model fitting, we wanted to ensure that the intercept terms in the model and regression-predicted costs by age using the models agreed to the annual expenditure in the data for individuals with none of the disease modelled. There was good correspondence of these predictions and those observed in the data for models including the disease comorbidity pairs; however, predictions for models not including the comorbidity pairs underestimated the costs among the nondiseased at younger ages (both OLS and gamma regressions) due to strong disease–disease–age interactions. Therefore, for models without comorbidity pair interactions, we rescaled all regression coefficients. First, we used the unscaled regression equations to predict costs among those with any disease and compared this with their actual total cost in the data set. We then used age-specific scalars for the disease coefficients in the model to return the correct total cost of people with diseases. For example, if the sum of OLS regression predicted that costs of 50–54-year-olds with at least one disease was $200 million, but the actual observed total cost for these same people was $180 million, then we rescaled all disease coefficients by 180/200 = 0.9 for this age group. Second, the reciprocal procedure was performed for people without disease. Notably, no such scaling was needed for the better-fitted models with the comorbidity pairs and their interactions, with age included.

Given age interactions, we present absolute estimates of disease expenditure for 60–64-year-olds as the main results, with young (45–49 years) and old (75–79 years) estimates presented additionally in **S4 Table** and **S5 Table**. Actual regression coefficients are presented in **S3 Table**, allowing interested readers to calculate excess costs for alternative groupings.

We present cause-deleted estimates, the estimates of spending without the presence of comorbidities, by setting disease coefficients in the predictive equation to zero.

We undertook several robustness checks. First, for simpler models (six-disease, no disease comorbidity pairs), an OLS general estimating equation (GEE) regression model on individual-level data was undertaken to allow for intraclass correlation due to up to 7 years of observation for each individual. Second, for simpler models we also ran (non-GEE) OLS regressions on unit-level data. Third, all the above models use between-person comparisons, which may be residually confounded by unobserved individual characteristics. Therefore, we also ran fixed effects models that capitalise on within-individual changes in disease status to estimate disease costs, removing potential time-invariant confounding [22].

## Results

There were 18.9 million person-year observations for those 25 years and older between 1 July 2007 and 30 June 2014. A total of 7.1 million (37.7%) of these observations included at least one NCD (**Table 1**). There was a total allocated expenditure across the 7 years of US$26.4 billion, of which $20.3 billion (77.0%) occurred among observations with at least one disease. Regarding source, half or $13.1 billion arose from inpatient care and about a fifth from each of outpatients and community pharmaceuticals.

**Table 1:** Descriptive data of observation counts and expenditure (US$ 2016) by sex, age, and financial year.

Variable	Counts			Expenditure (in US$ millions)		
	Without NCD	With NCD	Combined	Without NCD	With NCD	Combined
Total people observations and expenditure	11,775,499	7,127,885	18,903,384	$6,083	$20,331	$26,413
Expenditure by data source:^†^						
Inpatient				$2,214	$10,887	$13,101
Outpatient				$1,142	$4,103	$5,245
Pharmaceutical				$1,271	$3,763	$5,034
Laboratory				$924	$574	$998
Primary care				$1,024	$994	$2,018
People observations and expenditure by year:						
2007–2008	1,770,525	810,847	2,581,372	$856	$2,671	$3,528
2008–2009	1,748,868	882,412	2,631,280	$887	$2,549	$3,437
2009–2010	1,724,172	954,744	2,678,916	$925	$2,861	$3,786
2010–2011	1,687,564	1,024,697	2,712,261	$896	$2,948	$3,844
2011–2012	1,648,101	1,091,533	2,739,634	$887	$3,132	$4,018
2012–2013	1,613,234	1,155,265	2,768,499	$813	$3,066	$3,880
2013–2014	1,583,035	1,208,387	2,791,422	$818	$3,102	$3,919
Percentage distribution total observations by:						
Sex (female)	53.0%	53.3%	46.9%	60.9%	51.8%	46.1%
Age 25–44 years^‡^	49.7%	26.2%	40.8%	45.3%	14.9%	21.9%
Age 45–64 years	39.3%	38.5%	39.0%	35.6%	34.2%	34.5%
Age 65–74 years	7.5%	17.5%	11.2%	10.9%	23.2%	20.4%
Age 75–84 years	2.8%	12.7%	6.6%	5.9%	19.7%	16.5%
Age 85+ years	0.7%	5.2%	2.4%	2.2%	8.0%	6.6%

^†^Expenditure by data source is given by diseases in **S2 Table**.

^‡^Age at beginning of financial year.

The number of unique individuals contributing at least one person-year of observation was 3,223,929.

Abbreviation: NCD, noncommunicable disease.

**Table 2** shows the observations and total expenditure by disease phase. People with neurological conditions, musculoskeletal conditions, CVD, and diabetes had the greatest number of observations at 16.1%, 15.7%, 11.3%, and 7.5%, respectively, mostly as prevalent observations (i.e., neither diagnosed nor dying in that observation year). People with CVD had the highest total expenditure (43.1%), although this includes spending on additional diseases.

**Table 2:** Observation counts and expenditure (in US$ millions, 2016) by diseases, by phase, and by disease comorbidity combinations.

Disease variables	Person-year observations by disease phase				Percent^†^	Total expenditure by disease phase				Percent^†^
	Diag.	Prev.	Last	Any	Any	Diag.	Prev.	Last	Any	Any
Cancer	107,195	737,398	52,733	897,326	4.7%	$1,420	$3,167	$718	$5,305	20.1%
Lung cancer	7,139	9,671	10,387	27,197	0.1%	$114	$73	$126	$312	1.2%
Colorectal cancer	15,593	78,680	7,496	101,769	0.5%	$300	$400	$100	$800	3.0%
Breast cancer	17,588	181,907	3,928	203,423	1.1%	$218	$709	$56	$982	3.7%
Prostate cancer	19,268	173,573	3,964	196,805	1.0%	$114	$663	$43	$820	3.1%
Other cancer	47,607	293,567	26,958	368,132	1.9%	$675	$1,323	$393	$2,392	9.1%
CVD	220,595	1,874,070	35,962	2,130,627	11.3%	$2,523	$8,544	$328	$11,394	43.1%
IHD	87,634	884,231	20,109	991,974	5.2%	$931	$3,942	$174	$5,046	19.1%
Stroke	50,043	315,492	9,230	374,765	2.0%	$517	$1,382	$70	$1,969	7.5%
Other CVD	82,918	674,347	6,623	763,888	4.0%	$1,075	$3,220	$85	$4,380	16.6%
Type 2 diabetes mellitus (DM)	123,478	1,284,565	4,669	1,412,712	7.5%	$303	$4,640	$71	$5,014	19.0%
Chronic LLK disease	80,203	577,511	10,589	668,303	3.5%	$998	$3,397	$111	$4,508	17.1%
Chronic lung disease	37,577	259,395	9,088	306,060	1.6%	$454	$1,596	$84	$2,134	8.1%
Chronic kidney disease	27,281	203,890	585	231,756	1.2%	$308	$1,218	$16	$1,542	5.8%
Chronic liver disease	15,345	114,226	916	130,487	0.7%	$236	$583	$12	$832	3.2%
Neurological (Neuro)	434,123	2,600,585	13,316	3,048,024	16.1%	$2,713	$7,427	$57	$10,196	38.6%
Musculoskeletal (MS)	272,648	2,702,844	1,124	2,976,616	15.7%	$1,782	$8,354	$16	$10,151	38.4%
Cancer and CVD				189,605	1.0%				$1,562	5.9%
Cancer and DM				112,243	0.6%				$863	3.3%
Cancer and LLK				77,580	0.4%				$874	3.3%
Cancer and Neuro				263,464	1.4%				$2,549	9.7%
Cancer and MS				244,377	1.3%				$1,995	7.6%
CVD and DM				303,484	1.6%				$2,181	8.3%
CVD and LLK				210,282	1.1%				$2,071	7.8%
CVD and Neuro				536,303	2.8%				$3,893	14.7%
CVD and MS				665,981	3.5%				$4,415	16.7%
DM and LLK				121,131	0.6%				$1,167	4.4%
DM and Neuro				350,523	1.9%				$2,364	9.0%
DM and MS				439,640	2.3%				$2,528	9.6%
LLK and Neuro				240,226	1.3%				$2,212	8.4%
LLK and MS				261,819	1.4%				$2,365	9.0%
Neuro and MS				875,633	4.6%				$5,046	19.1%

^†^For person-year, percentage of 18.9 million person-year observations (**Table 1**); for expenditure, percentage of $26,413 million (**Table 1**).

Disease groups are not mutually exclusive, i.e., a single person-year observation with diagnoses of CVD, DM, and MS will contribute observations (and expenditure) to the three separate diseases, in one (only) of diag., prev., and last; and to CVD and DM, CVD and MS, and DM and MS disease combinations.

Abbreviations: CVD, cardiovascular disease; DM, type 2 diabetes mellitus; Diag., first year of diagnosis; IHD, ischaemic heart disease; Last, last year of life and dying of this disease; LLK, lung/liver/kidney; MS, musculoskeletal; Neuro, neurological; Prev., prevalent years.

### Excess disease costs per person

**Table 3** shows the modelled health system expenditure per person-year for 60–64-year-olds, attributed to each disease phase, for both the aggregated and disaggregated-disease models, by sex, without and with disease comorbidity pairs. The first row of the table results (‘No NCD’) is the estimated expenditure for a 60–64-year-old with no NCDs; the next panel is the main effects by disease phase for each of the 6 or 13 diseases; and the last panel is the expenditure per year for the 15 comorbidity pairings, over and above that attributed to the diseases independently.

**Table 3:** Annual excess health spending (US$ 2016) for NCDs (6 and 13 disease groupings) predicted by OLS regression for 60–64-year-olds†.

Variable	Males				Females			
	Aggregated: 6 diseases		Disaggregated: 13 diseases		Aggregated: 6 diseases		Disaggregated: 13 diseases	
	MA: Base model, scaled	MAC: Disease interactions	MD: Base model, scaled	MDC: Disease interactions	FA: Base model, scaled	FAC: Disease interactions	FD: Base model, scaled	FDC: Disease interactions
No NCD	556	613	553	589	626	633	624	616
DISEASE PHASE MAIN EFFECTS (for 62.5-year-olds, as age [centred on 62.5 then divided by 10] and age squared; both interacted with disease in all models)								
Disease main effects—first year of diagnosis								
Cancer	7,773	8,515			9,747	10,915		
Lung			10,518	11,539			10,809	11,680
Colorectal			14,014	15,884			13,660	15,406
Breast							8,216	9,123
Prostate			2,759	2,808				
Other			10,445	11,514			9,532	10,481
CVD	7,519	8,417			5,978	6,443		
IHD			7,264	8,176			5,267	5,665
Stroke			5,175	5,616			5,835	6,328
Other CVD			8,451	9,617			6,714	7,494
DM	443	78	445	87	261	−10	263	−5
Chronic LLK	7,181	6,377			6,654	6,348		
Chronic lung			7,008	6,443			5,979	5,715
CKD			6,546	6,111			6,841	6,844
CLD			8,313	8,064			7,621	7,698
Neurological (Neuro)	5,110	4,851	4,930	4,606	2,991	2,956	2,958	2,888
Musculoskeletal (MS)	3,275	3,522	3,273	3,491	4,647	5,148	4,671	5,107
Disease main effects—last year of life if dying of disease								
Cancer	9,191	8,784			9,815	9,853		
Lung			6,980	6,618			7,646	7,286
Colorectal			9,519	9,541			9,815	9,915
Breast							9,603	9,568
Prostate			7,699	6,911				
Other			10,358	10,292			10,826	10,934
CVD	8,111	8,397			8,825	9,225		
IHD			5,818	5,932			6,879	6,866
Stroke			5,196	5,396			6,705	7,323
Other CVD			11,361	12,654			11,131	12,333
DM	14,251	14,234	13,798	13,720	16,042	16,892	15,674	16,535
Chronic LLK	9,076	8,242			9,252	9,051		
Chronic lung			7,009	6,360			7,198	6,913
CKD			21,724	22,461			28,269	31,241
CLD			7,385	6,846			8,612	8,930
Neuro	3,825	3,642	3,897	3,689	3,310	3,458	3,385	3,504
MS	16,072	17,864	15,896	17,575	12,819	14,299	12,607	13,944
Disease main effects—prevalent years of diagnosis								
Cancer	1,710	1,210			1,716	1,296		
Lung			3,207	2,781			3,601	3,043
Colorectal			2,686	2,422			2,042	1,613
Breast							1,320	912
Prostate			723	357				
Other			2,211	1,843			1,701	1,264
CVD	1,451	1,007			1,439	839		
IHD			977	716			1,117	673
Stroke			792	382			790	304
Other CVD			1,393	1,252			1,430	1,202
DM	1,122	614	1,110	616	1,018	706	1,013	711
Chronic LLK	2,936	1,052			2,541	1,291		
Chronic lung			2,415	806			2,229	1,077
CKD			4,276	3,209			3,379	2,767
CLD			1,674	261			1,555	652
Neuro	1,329	363	1,307	375	860	385	854	393
MS	756	435	730	442	1,084	806	1,074	803
DISEASE COMORBIDITY INTERACTIONS (for 62.5-year-olds, as age [centred on 62.5] and age squared; both interacted with diseases in models) (95% CI)^‡^								
Cancer and CVD		−246 (−517, 25)		−484 (−728, −240)		−25 (−281, 231)		−124 (−366, 118)
Cancer and DM		21 (−286, 328)		−90 (−367, 187)		16 (−219, 251)		49 (−167, 265)
Cancer and LLK		277 (−82, 636)		−249 (−573, 75)		366 (71, 661)		178 (−97, 453)
Cancer and Neuro		3,160 (2,947, 3,373)		2,786 (2,593, 2,979)		2,435 (2,282, 2,588)		2,327 (2,184, 2,470)
Cancer and MS		1,045 (840, 1,250)		1,090 (904, 1,276)		732 (549, 915)		803 (633, 973)
CVD and DM		1,074 (901, 1,247)		936 (780, 1,092)		1,026 (853, 1,199)		907 (745, 1,069)
CVD and LLK		2,498 (2,264, 2,732)		2,050 (1,837, 2,263)		2,456 (2,238, 2,674)		2,131 (1,925, 2,337)
CVD and Neuro		1,001 (849, 1,153)		933 (796, 1,070)		852 (720, 984)		724 (601, 847)
CVD and MS		654 (522, 786)		431 (315, 547)		651 (509, 793)		473 (341, 605)
DM and LLK		1,343 (1,092, 1,594)		1,160 (933, 1,387)		909 (696, 1,122)		697 (499, 895)
DM and Neuro		1,689 (1,523, 1,855)		1,702 (1,552, 1,852)		835 (710, 960)		844 (728, 960)
DM and MS		401 (263, 539)		424 (299, 549)		295 (162, 428)		315 (192, 438)
LLK and Neuro		1,726 (1,513, 1,939)		1,570 (1,379, 1,761)		1,038 (875, 1,201)		872 (720, 1,024)
LLK and MS		1,730 (1,530, 1,930)		1,404 (1,224, 1,584)		1,139 (968, 1,310)		983 (824, 1,142)
Neuro and MS		795 (673, 917)		816 (706, 926)		737 (644, 830)		756 (668, 844)

^†^Regression coefficients for OLS models are shown in **S3 Table**. Excess costs for 45–49- and 75–79-year-olds are shown in **S4 Table** and **S5 Table**.

^‡^CIs (95%) are shown only for the disease comorbidity pairs; the vast majority of main effects had 95% CI excluding the null.

Scaling required for models without disease–disease interactions is described in Materials and methods.

Abbreviations: CI, confidence interval; CKD, chronic kidney disease; CLD, chronic liver disease; CVD, cardiovascular disease; DM, diabetes; FA, females aggregated diseases model with no comorbidity pair interactions; FAC, females aggregated-disease model with comorbidity pair interactions; FD, females disaggregated diseases model with no comorbidity pair interactions; FDC, females disaggregated-disease model with comorbidity pair interactions; IHD, ischaemic heart disease; LLK, lung/liver/kidney; MA, males aggregated diseases model with no comorbidity pair interactions; MAC, males aggregated-disease model with comorbidity pair interactions; MD, males disaggregated diseases model with no comorbidity pair interactions; MDC, males disaggregated diseases model with comorbidity pair interactions; MS, musculoskeletal; NCD, noncommunicable disease; Neuro, neurological; OLS, ordinary least squares.

When considering the aggregated-disease model for females, without comorbidity pairs (FA model in **Table 3**), a 60–64-year-old without any of the six diseases was estimated to cost the health system US$626 per year. If she had a cancer diagnosed in that year, her costs were predicted to increase by $9,747 over and above the $626 base cost, by $9,815 if dying of cancer in that year, or by $1,716 if living with a prevalent cancer. If she also had prevalent CVD, her costs were predicted to increase a further $1,439.

**Fig 1:**
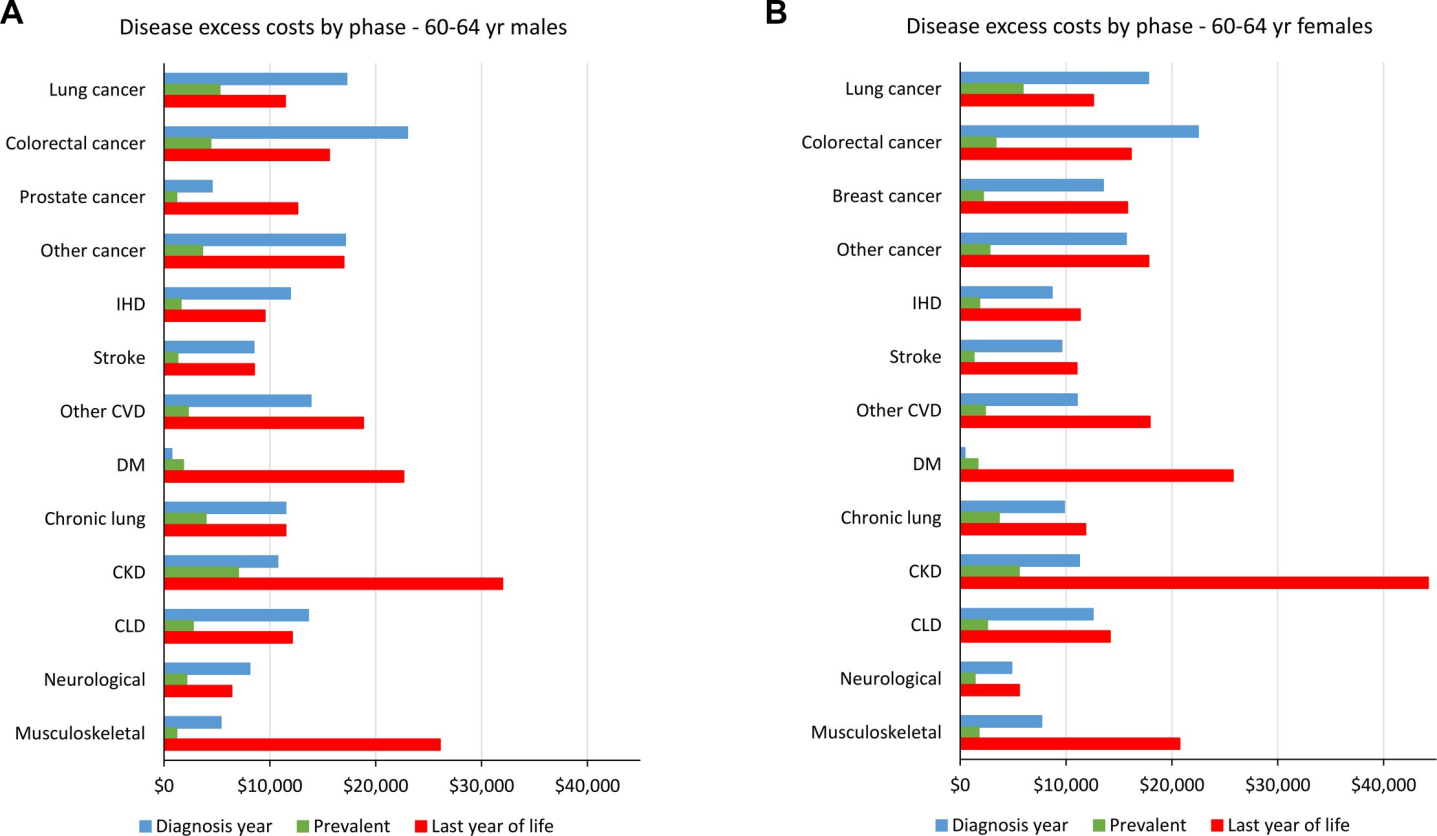
Independent disease expenditure per annum per person by disease phase (i.e., mutually exclusive between diseases, not allowing for disease comorbidity interactions; US$ 2016). CKD, chronic kidney disease; CLD, chronic liver disease; CVD, cardiovascular disease; IHD, ischaemic heart disease.

**Fig 1** shows these independent excess costs for both male and female 60–64-year-olds, for the disaggregated-disease models (i.e., estimates for the MD and FD model columns of **Table 1**). Excess costs in the year of diagnosis and year of death from that disease were higher than prevalent costs. However, patterns differed by disease. For example, cancer and CVD excess costs in the year of diagnosis and last year of life were roughly similar, but diabetes costs if dying of diabetes ($13,798 and $15,674, **Table 1**) were much higher than costs in the diagnosis year. Last year of life costs for chronic kidney disease were also notably high.

Excess costs for the disease comorbidity pairs were positive, with 95% confidence interval (CI) excluding the null for all pairs across all models, except for cancer with each of CVD, diabetes, and LLK disease (bottom panel of **Table 3**). That is, the costs of having diseases jointly was usually greater than the estimated summed costs for having the diseases independently (shown in the rest of **Table 3**). For example, using the male disaggregated-disease model (model MDC in **Table 3**), a 60–64-year-old with both CVD and LLK disease was predicted to cost $2,050 (95% CI $1,837–$2,263) more than that predicted based on the independent excess costs due to CVD and LLK independently. Only 1 of the 60 comorbidity pairs (15 possible pairs across four models) had a negative cost with the 95% CI excluding the null, namely the cancer-CVD pair in the male disaggregated-disease model (−$484, 95% CI −$784 to −$240). Whilst this in isolation may be a chance finding given the 60 pairs tested, this same cancer-CVD pair had a negative cost for the male aggregated-disease model and female aggregated and disaggregated models—but with 95% CI including the null.

**Fig 2:**
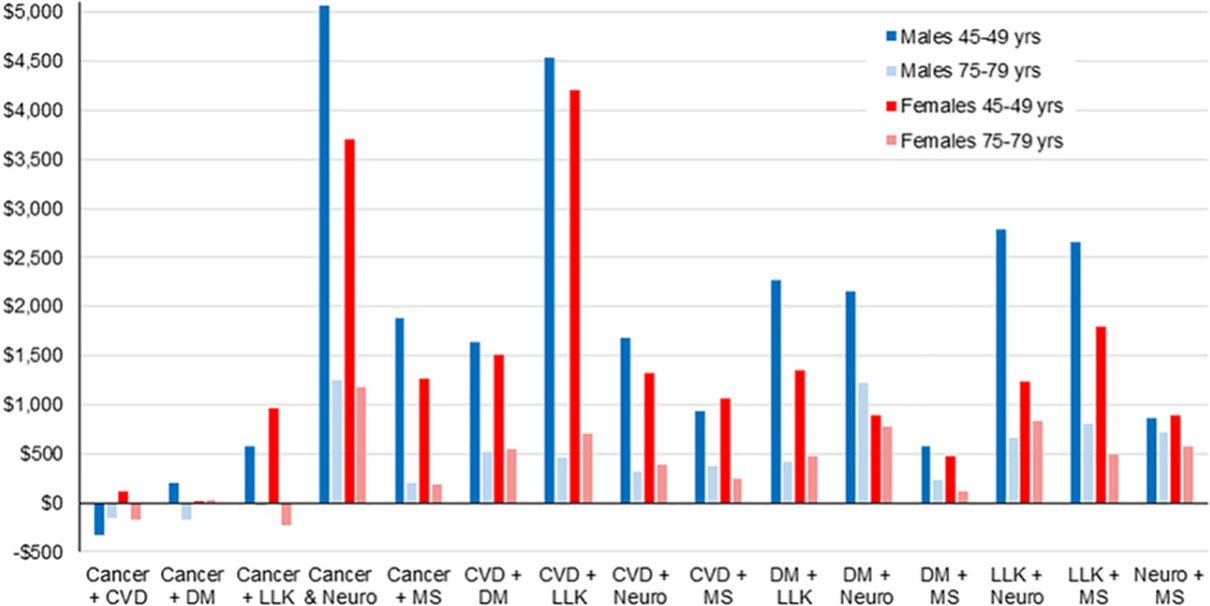
Estimated excess costs per annum for 15 disease comorbidity pairs for 45–49- and 75–79-year-olds, using the six-disease models in Table 3. CVD, cardiovascular disease; DM, diabetes mellitus; LLK, lung/liver/kidney; MS, musculoskeletal; Neuro, neurological.

There were significant interactions of age and age squared with most separate diseases and disease comorbidity interactions (**S3 Table**). Because of these age interactions, excess costs vary by age (**S4 Table** and **S5 Table** give estimates for 45–49- and 75–79-year-olds, respectively). Two patterns were evident for independent disease costs: excess costs in the last year of life were less with increasing age, and musculoskeletal and neurological excess disease costs per person in the first year of diagnosis and prevalent cases were greater for 75–79-year-olds than 45–49-year-olds. Regarding the disease comorbidity pairs, there was a strong pattern of comorbidity costs being greater for 45–49-year-olds than 75–79-year-olds—often considerably so (**Fig 2**). For example, excess expenditure for 45–49-year-old males with CVD and chronic LLK was 10 times higher than for 75–79-year-olds, and six times higher for females. High excess costs for comorbidity are notable for cancer and neurological, and CVD and LLK (especially at younger ages).

### Disease costs at population level

**Fig 3:**
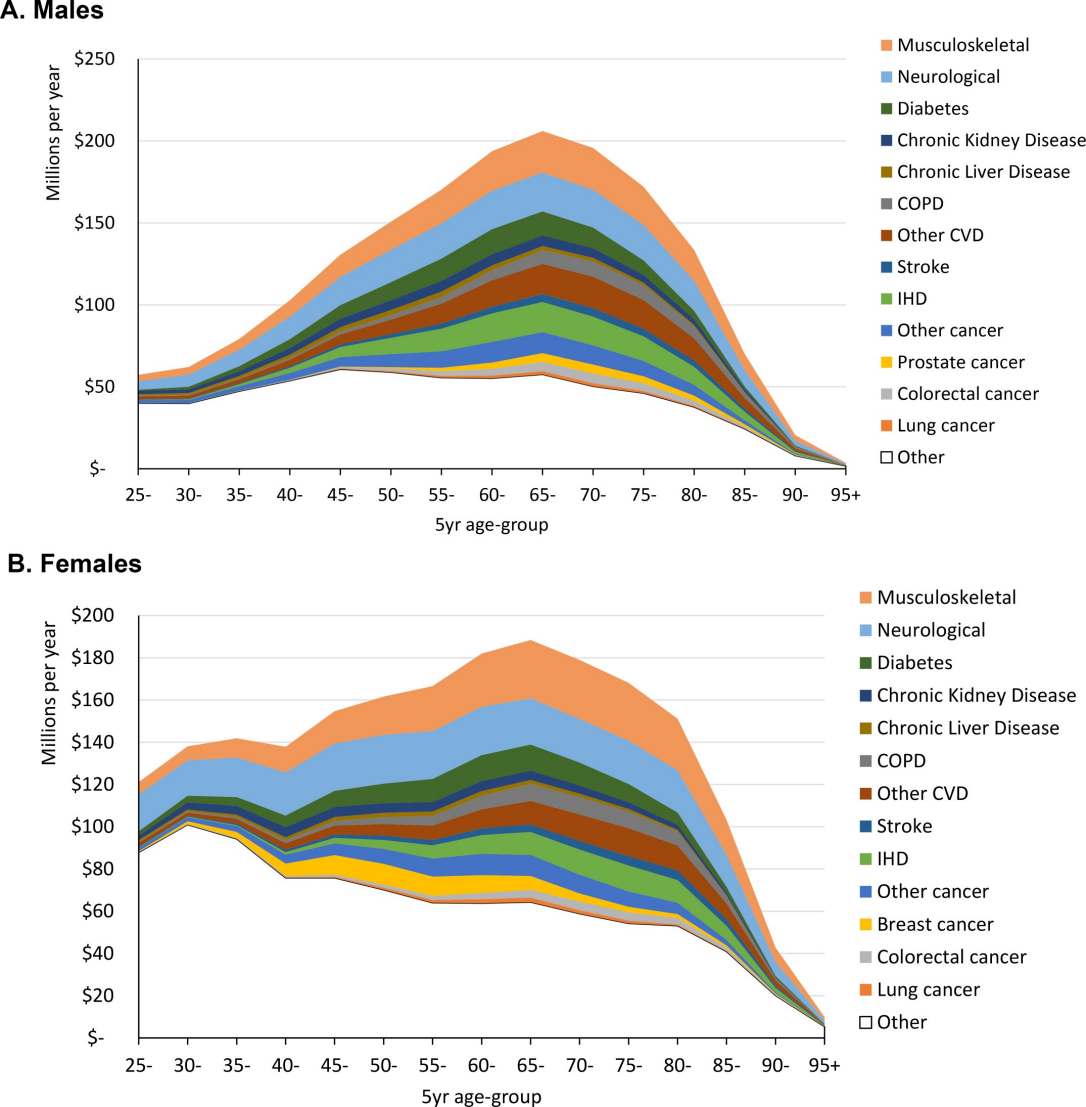
**Stacked line graph of cumulative health system expenditure of 13 diseases (US$ 2016) by age, assuming disease independence: (a) males, (b) females.** COPD, chronic obstructive pulmonary disease; CVD, cardiovascular disease; IHD, ischaemic heart disease.

Total expenditure by disease costs is a function of the above per-person costs and how common the disease phases actually were (**Table 2**). Across the 7 years in the study, using the models without comorbidity interactions, the greatest excess expenditures were for neurological (22.3%), CVD (21.2%), and musculoskeletal (20.8%) disease (**Table 4**). **Fig 3** shows the attribution of this disease expenditure by sex and age. A greater area under the curve for females is attributed to nonmodelled diseases at younger ages, consistent with maternity care not being included as an explicit category in the modelling. Noteworthy patterns include that neurological and musculoskeletal costs were substantial at all ages, with neurological particularly notable at younger ages for females; diabetes costs and female breast cancer costs were skewed more to younger ages; and other CVD and IHD costs were considerably greater than stroke costs at all ages.

**Table 4:** Population-level total costs and cause-deleted cost savings in absolute dollars (US$ millions) and as a percentage of cost savings from deleting all diseases.

Total health expenditure	Model A: Main effects (no comorbidity pairs)			Model B: Including 15 comorbidity pairs			Ratio of expenditure attributed to diseases with comorbidity expenditure separated out (model B) compared with not (model A)
	$26,413			$26,413			
Total NCD expenditure (i.e., cost savings from deleting all NCDs)	$15,596			$15,455			
Disease	Expenditure	Percentage of total NCD expenditure	Percentage of total health expenditure	Expenditure	Percentage of total NCD expenditure	Percentage of total health expenditure	
Cancer	$2,354	15.1%	8.9%	$2,175	14.1%	8.2%	0.92
CVD	$3,311	21.2%	12.5%	$2,889	18.7%	10.9%	0.87
Chronic LLK disease	$1,798	11.5%	6.8%	$1,137	7.4%	4.3%	0.63
Diabetes	$1,411	9.0%	5.3%	$846	5.5%	3.2%	0.60
Neurological	$3,484	22.3%	13.2%	$2,219	14.4%	8.4%	0.64
Musculoskeletal	$3,239	20.8%	12.3%	$2,509	16.2%	9.5%	0.77
**Sum**	**$15,596**	**100.0%**	**59.0%**	**$11,775**	**76.2%**	**44.6%**	
15 comorbidity pairs				$3,680	23.8%	13.9%	
**Sum**	** **	** **		**$15,455**	**100%**	**58.5%**	

Uses the aggregated six-disease models above in Table 3.

The percentage distribution for the 13 disaggregated diseases not presented here are: lung cancer 1.0% of total NCD expenditure (0.6% of all expenditure); colorectal cancer 2.6% (1.5%); breast cancer 2.8% (1.7%); prostate cancer 1.2% (0.7%); IHD 9.0% (5.3%); stroke 3% (1.8%); COPD 4.9% (2.9%); chronic liver disease 2.0% (1.2%); and chronic kidney disease 4.8% (2.8%).

Abbreviations: COPD, chronic obstructive pulmonary disease; CVD, cardiovascular disease; IHD, ischaemic heart disease; LLK, lung/liver/kidney; NCD, noncommunicable disease.

Using the models with the 15 comorbidity pairs, the independent disease costs reduced by 23.8% due to costs now being attributed to one of the 15 possible comorbidity pairs (Table 4). Of this 23.8% comorbidity expenditure, the five comorbidity pairs including a neurological condition contributed 60.7%, and the five pairs including musculoskeletal contributed 41.4%. The comorbidity pair with the greatest contribution was neurological plus musculoskeletal (18.1%), meaning the 9 out of 15 comorbidity pairs including either/both neurological and musculoskeletal conditions explained 84.0% of additional health expenditure due to comorbidities at the population level (i.e., 60.7% + 41.4% − 18.1%; see **S1 Fig** for a breakdown of percentage comorbidity expenditure for each of the 15 comorbidity pairs).

Of the 76.2% of expenditure not captured by comorbidity pairs, CVD accounted for 18.7%, followed by musculoskeletal (16.2%), neurological (14.4%), cancer (14.1%), chronic LLK disease (7.4%), and diabetes (5.5%; Table 4). The expenditure attributed to these six diseases independently, when including comorbidity pairs relative to the model excluding comorbidity pairs, varied by disease, ranging from 0.60 for diabetes (i.e., 40% of apparent independent expenditure for diabetes can actually be attributed to superadditive costs arising from diabetes coexisting with other diagnoses) to 0.92 for cancer (i.e., only 8% of apparent independent expenditure on cancer can be attributed to comorbidity additive effects; last column of Table 4).

### Sensitivity analyses

The coefficients across the preferred aggregated data OLS models and the unit-level OLS and GEE unit-level OLS regressions were identical (**S6 Table**). The standard errors were smallest for unit-level OLS but more similar for aggregate OLS and GEE unit level. However, given the size of the data sets and the usually sizeable health expenditures for diseases, most disease excess costs are statistically significant, regardless. Fixed effects analyses that use within-person variation (i.e., differences in expenditure between years, with and without disease, or with varying disease phase) were in reasonable agreement with our preferred OLS regressions on aggregate data (**S2 Fig**), given conceptual differences between the approaches and data limitations (see **S1 Appendix**).

## Discussion

### Main findings and interpretation

**Fig 4:**
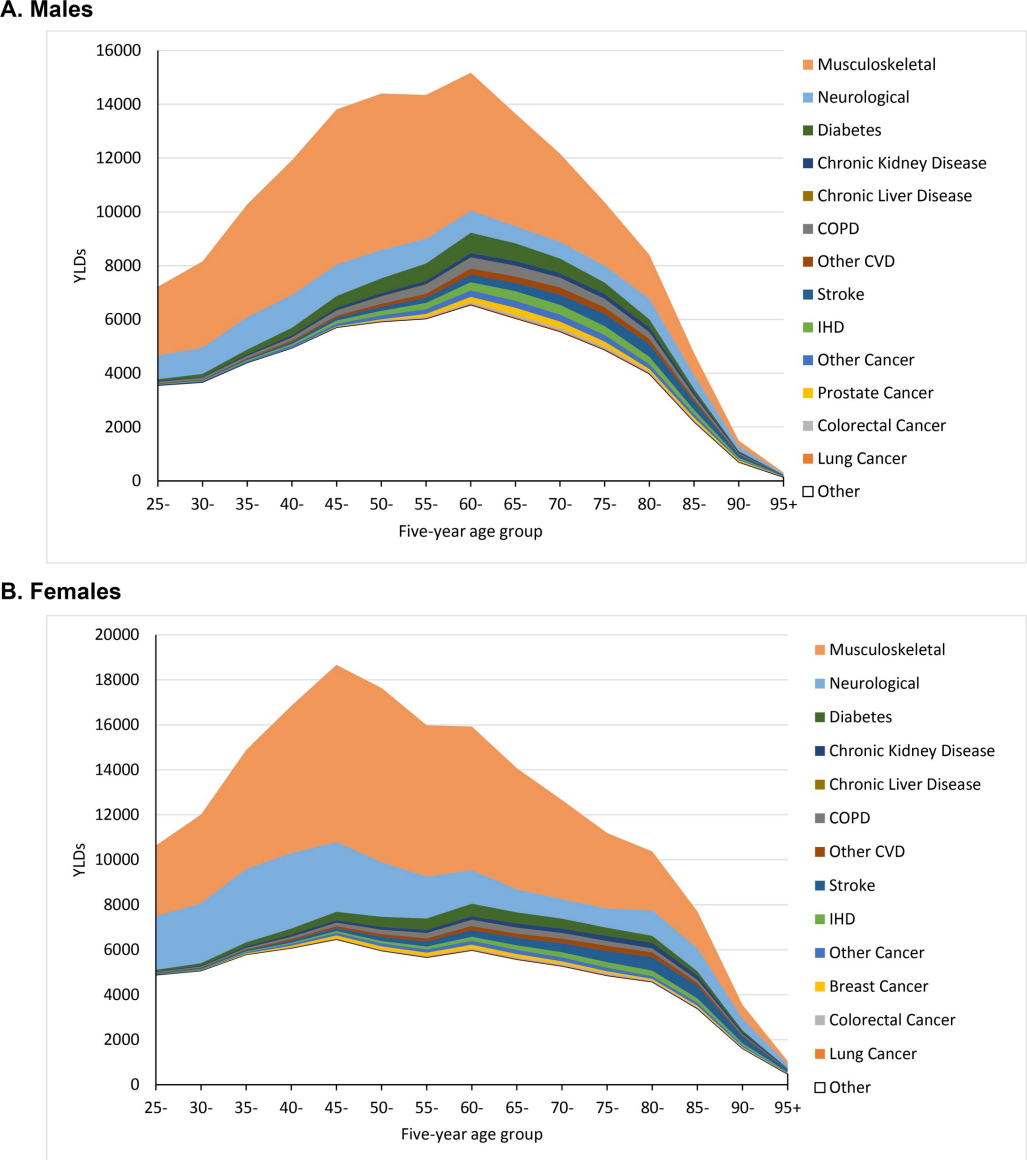
**Stacked line graph of cumulative YLDs for 13 diseases by age according to the GBD 2016, for the same disease groupings used for expenditure in Fig 3: (a) males, (b) females.** COPD, chronic obstructive pulmonary disease; CVD, cardiovascular disease; GBD, Global Burden of Disease; IHD, ischaemic heart disease; YLD, year of life with disability. *Source: GBD 2016 [23]*.

This study estimates health system expenditure for many NCDs, by disease phase, for New Zealand. We capitalised on linked health data with unique identifiers for the entire population that track medical costs over multiple years. We used a regression approach to estimate the excess cost to the health system from having a given disease. At the population level, we found that 59% of all allocated health system expenditure was attributable to the included NCDs. Attributing costs to separate disease groupings and not yet allowing for comorbidities, neurological diseases accounted for 22.3% of NCD expenditure, followed by CVD (21.2%), musculoskeletal (20.8%), cancer (15.1%), chronic LLK disease (11.5%), and diabetes (9.0%). We were surprised by large costs due to neurological and musculoskeletal diseases; therefore, we plotted in **Fig 4** years of life lived with disability in New Zealand by sex, age, and disease, from GBD data using the same disease categorisations for New Zealand, paralleling our **Fig 3** for costs. Other studies have found a correlation of health expenditure with years of life with disability (YLDs) [9,10], although the correlation is even stronger with disability-adjusted life years. The New Zealand neurological and musculoskeletal morbidity burdens stand out even more for YLDs (**Fig 4**) than for costs (**Fig 3**), offsetting possible concerns that our costings for neurological and musculoskeletal disease might be too high.

How does this distribution of costs, ignoring comorbidities for now, compare with previous studies? There is a body of work in the US using methods that attributed events to diseases—not an excess costing approach using regression. For example, Dieleman and colleagues (2016) attributed 16 years of health expenditure using multiple data sources in the US and found that CVD contributed 11% of all health expenditure (12.5% in our New Zealand study, using the total health expenditure as the denominator in Table 4), cancers 5.5% (New Zealand = 8.9%), COPD 2.6% (4.9%), neurological 4.8% (13.2%), musculoskeletal 8.7% (12.3%), and diabetes 4.8% (5.3%) [8]. In another comparable study using a GBD grouping of diseases for Switzerland, Wieser and colleagues (2018) found that cancer contributed 6.0%, CVD 15.6%, diabetes 1.5%, neurological (including dementia) 3.7%, and musculoskeletal 13.4% [9]. Musculoskeletal expenditure in New Zealand, at 12.3%, is intermediary between these US and Swiss percentage estimates. Of note, in GBD 2016, New Zealand also had 1.31 times higher morbidity burden for back pain and 1.84 times higher ‘other’ musculoskeletal than expected based on its level of sociodemographic development [23]. This leaves the neurological category as the notable ‘stand-out’ high cost for New Zealand, perhaps due to both the range of disease and conditions included (e.g., epilepsy, dementia, migraine) and the inclusive capture of people with at least one neurological diagnosis (e.g., through the range of data sets and classifications; **S1 Table**).

Where our New Zealand study steps ahead of similar general COI studies is in further disaggregating disease costs by phase and comorbidities, an advance that is possible due to all health data sets being linked at the individual level. Disease costs were generally higher in the year of diagnosis and the year of death if dying of that disease (**Fig 1**), consistent with our previous work on multiple cancers [24]. Costs in the year of death if dying from chronic kidney disease, musculoskeletal, or diabetes were particularly prominent, ranging from $13,000 to $30,000 (**Fig 1**). Dying-related costs from kidney disease, if also undergoing (failed) transplant or renal dialysis, are high. Dying of diabetes per se, whilst uncommon compared with dying of diseases elevated in risk by diabetes, is also expectedly expensive.

Regarding comorbidities, we find that the majority of comorbidity pairs increase costs more than that expected based on having the same diseases separately (Table 3, **Fig 1**), and this superadditive comorbidity cost was far more prominent at younger ages (**Fig 2**). Whilst we have no direct evidence, it seems plausible that young people with comorbidities might be treated more aggressively than old people with comorbidities or perhaps that young people with comorbidities have more severe disease than their younger counterparts with just one disease. Further research to understand this this age variation in comorbidity expenditure is warranted.

We estimated that 23.8% of all NCD health expenditure is attributable to these additional increments of comorbidity, over and above the independent costs of having diseases separately. Most previous COI studies analysing the impact of comorbidities have been from the perspective of one disease only, and its increased costs due to comorbid conditions (e.g., [25–27]). An exception is Dieleman and colleagues (2017), who used encounter-level data with multiple ICD codes recorded for each event, to quantify ‘inflows’ and ‘outflows’ between all comorbidities, to reallocate health spending mutually exclusively [12]. This is a different approach to what we took using a person-based excess costing approach to directly examine disease–disease interactions based on diagnoses stated on any event linked for the same person, and quantify the independent contributions of separate diseases and comorbidity pairs to total health expenditure. We found that the majority of the 15 comorbidity pairs increased expenditure over and above having the diseases separately. There was, however, evidence that having cancer and CVD simultaneously reduced health expenditure compared to that expected from having the two diseases separately. It is possible that a diagnosis of cancer means that treatments for other diseases are down-prioritised, but this is speculation on our part.

### Strengths and limitations

There are limitations with our study. First, cost weights assigned to events may not have been the exact price to hospitals or other fund managers. Second, whilst New Zealand has high-quality linked heath data, it does not capture all costs. Private expenditure is estimated at 18% of all health expenditure; future research could attempt to include this but will require additional data and assumptions about how this funding is distributed compared with public funding. For example, the great majority of NCDs are treated in public hospitals, but breast cancer treatment and hip/knee replacement surgery are more commonly provided in the private sector. There are also missing costs due to support care services (e.g., rest home care of people with dementia). We were unable to capture actual primary care usage by individuals on national databases; rather, we just imputed an ‘expected’ cost based on the bulk funding formula, and we will thus have underestimated disease costs somewhat, as people with the diseases we captured will (presumably) be higher users of primary care. We also do not include prevention costs. Nevertheless, we capture the majority of costs, and certainly enough costs to confidently speak to patterns of cost by sex, age, disease, disease phase, and comorbidity pairs. Future research should aim to improve these data, however.

Third, whilst we see the excess costing approach using regression modelling as a strength overall, and it avoids the complexities of attributing each individual medical event to a disease, it has limitations. People with diseases may be higher users of health services, regardless of their disease—a form of time-invariant confounding. In fixed effects analyses exploiting changes in costs within individuals (see **S1 Appendix**), we found broadly similar costs, suggesting that time-invariant confounding is not too problematic. There may also be time-varying confounding, whereby people with a disease increase health services use for other reasons, including increasing surveillance and preventive activities when engaged with the health system. Fourth, addition of further comorbidity combinations (e.g., with mental illness) would likely increase the proportion of all NCD health expenditure attributable to the superadditive effect of comorbidities.

### Potential implications for researchers, planners, and policy makers

So what for researchers and policy makers? There is a surprising lack of disease-attributed costing studies involving multiple diseases at once. Governments and health systems managers and funders can improve planning and prioritisation, knowing where the money goes. Also, cost-effectiveness studies usually need costs by disease to model cost offsets from preventing disease (presentations) and, conversely, the health systems costs from living longer in evaluations using an unrelated disease costing approach [28,29]. Our analytical framework generates these costs by disease phase for such cost-effectiveness modelling (e.g., [30]). We believe the costing methods used in this paper can be applied elsewhere. For example, the OECD has as a goal that national health expenditure be disaggregated by disease; this will require standardisation as best as possible of data and methods across countries, but given data variations across countries, exact standardisation is unlikely. We therefore propose that an approach similar to that used for estimating epidemiological parameters in the GBD may be useful [2,31]. Here, one would build up from COI studies in multiple countries (e.g., [5]) and use country-level parameters such as GPD, health system configuration, and such to build a model predicting disease costs within an envelope of total health expenditure given by a system of national health accounts. Our paper and methods may help in that the regression models built within a given (or few) country predict the relative expenditure by disease—which could be merged with the population demography, disease epidemiology, and total health expenditure of another country to at least provide an initial costing.

Finally, any national costing study will be dependent on the data available and methods used. That said, we suspect that the general patterns of these results (i.e., of comorbidity impacts being superadditive) for New Zealand are likely to be generalisable to other high-income countries. The methods we applied in this paper could be applied elsewhere to test for such generalisability—or not—of comorbid costs.

## Supporting information

S1 Appendix Analysis plan, OLS regression equations, and comparison of OLS and fixed effects. OLS, ordinary least squares.
(DOCX)

S1 RECORD Statement RECORD checklist. (DOCX)

S1 Table Disease and condition case definitions. (DOCX)

S2 Table Total expenditure (NZ$ 2011) for people with diseases and disease comorbidity combinations, by data source (not mutually exclusive between diseases, but mutually exclusive between sources). (DOCX)

S3 Table Additional regression coefficients (and s.e.; NZ$ 2011) for the age-by-disease, age-squared-by-disease, and age-by-disease-by-disease interactions. s.e., standard error. (DOCX)

S4 Table Annual excess health spending (US$ 2016) for NCDs (6 and 13 disease groupings) predicted by OLS regression for 45–49-year-olds. NCD, noncommunicable disease; OLS, ordinary least squares.
(DOCX)

S5 Table Annual excess health spending (US$ 2016) for NCDs (6 and 13 disease groupings) predicted by OLS regression for 75–79-year-olds. NCD, noncommunicable disease; OLS, ordinary least squares.
(DOCX)

S6 Table Comparison of coefficients (s.e. in parentheses; NZ$ 2011) for three modelling approaches, for six-disease model without comorbidity interactions: OLS regression on aggregated data (default method, as shown in Table 3), OLS regression on individual-level data, and OLS regression on individual-level data with GEE. GEE, general estimating equation; OLS, ordinary least squares; s.e., standard error.
(DOCX)

S7 Table Comparison of coefficients (s.e. in parentheses; NZ$ 2011) for OLS regression on individual-level data (as shown in S6 Table) compared with fixed effects regression on individual-level data utilising within-person changes by year in disease status and health expenditure. OLS, ordinary least squares; s.e., standard error.
(DOCX)

S1 Fig The percentage distribution of the comorbidity expenditure at the population level for the 15 comorbidity pairs. (TIF)

S2 Fig Comparison of between-person (OLS regression on aggregate data, as shown in Table 3) and within-person (OLS fixed effects) approaches. Coefficients are in NZ$ 2011. OLS, ordinary least squares.
(TIF)

## Abbreviations

CI: confidence interval
COI: cost of illness
CVD: cardiovascular disease
GBD: Global Burden of Disease
GEE: general estimating equation
ICD: International Classification of Disease
IHD: ischaemic heart disease
LLK: lung/liver/kidney
NCD: noncommunicable disease
NMDS: National Minimum Data Set
NZBDS: New Zealand Burden of Disease Study
OLS: ordinary least squares
YLD: year of life with disability
