# Supplementary material for: Health system costs for individual and comorbid noncommunicable diseases: An analysis of publicly funded health events from New Zealand
Source: PLoS Med. 2019 Jan 8;16(1):e1002716. doi: 10.1371/journal.pmed.1002716 (PMC6324792; doi:10.1371/journal.pmed.1002716)
Supplement: S1 Appendix — OLS, ordinary least squares. (DOCX) [file pmed.1002716.s001.docx]

# S1 Appendix

## **Analysis plan: a priori and iterative**

For clarity of research process, we briefly describe here the elements of our analysis that were a priori and remained to the final analyses in this paper, and those parts of the final analysis that were not as envisaged at the outset.

The following were a priori decisions, persisting through to the results as presented:

- The decision to use an excess or net costing approach
- The choice of six and 15 disease groupings, following GBD groupings used in the New Zealand Burden of Disease study
- The focus on comorbidity costs for the six disease groups (given 15 comorbidity dyads for 6 separate diseases, versus an wieldy 15!/([15-2]!×2!) = 105 disease parings for the 13 disease model.
- The attribution of diseases to phases (i.e. first year of diagnosis, prevalent and last year of life if dying of the disease) (but see below for comorbidity pairs).

Changes to the initial analysis plan, and the reasons why, are:

- We initially intended to use fixed effects as the main regression method. However, we became concerned that the date of diagnosis was not good enough to demarcate transitions from health to disease within individuals – the transition about which a within person method such as fixed effects relies upon (see further text in main paper; and fixed effects analyses are presented in supplementary material). Accordingly, we changed to using between person regression approaches as the preferred method.
- Among the between person approaches, we had anticipated using gamma regression as the main approach. However, it suffered from the following problems: when using the expenditures predicted by Gamma regression, the sum of these across all observations did not return the actual total health expenditure (whereas OLS regression did); and being a log-link model, the coefficients were not as directly interpretable as those from OLS. OLS therefore became our default method (although some Gamma and GEE regression (between and within person) results are included in supplementary material for interested readers).
- We had not anticipated at the outset that the models for diseases separately (i.e. without disease-disease pairings) would fail to predict non-disease person expenditure, even though total expenditure was predicted accurately. Given the non-diseased person’s expenditure is the base costs that our excess costs refer to, this is an important entity to predict. The reason for this poor model fit was the absence of disease-disease-age interactions; these were included in our model with comorbidity pairs, with good model fit. Statistically, therefore, one could argue our regression models without disease-disease comorbidity pairs are ‘invalid’. However, we disagree as it is a common (and we think valid) research question to estimate the costs of diseases separately – putting aside comorbidity. Accordingly, we developed the scalars as described in the text for the models without disease-disease and disease-disease-age interactions.
- We had planned to fit comorbidity pairs including disease phase (e.g. first year of cancer + prevalent CVD). However, this lead to an explosion of regression coefficients 8×15 = 120 comorbidity dummies (plus age interactions) rather than ‘just’ the 15 comorbidity pairs. We therefore abandoned this approach, although we note that shrinkage and other methods to address this issue in future research may be fruitful.

## **OLS regression model equation**

The OLS regression model equation for the six disease model with disease comorbidity pairs is shown below:

$$Total expenditure in each person year= \alpha+\theta_{1}.Age+ \theta_{2}.Age\_Sq + \theta_{3}.Year+\sum_{j=1}^{6} \sum_{k=0}^{3} \beta_{j,k}{.Dis\_Phase}_{j,k}+ \sum_{j=1}^{6} \sum_{k=0}^{3} \gamma_{j,k}{.Dis\_Phase}_{j,k}.Age+\sum_{j=1}^{6} \sum_{k=0}^{3} \delta_{j,k}{.Dis\_Phase}_{j,k}.Age\_Sq + \sum_{j=1}^{6} \sum_{l=j+1}^{6} \varphi_{i,j}.{Dis}_{i}.{Dis}_{l}+ \sum_{j=1}^{6} \sum_{l=j+1}^{6} \omega_{i,j}.{Dis}_{i}.{Dis}_{l}.Age+\epsilon$$

Where the variables are:

- Age is a continuous variable for age centered on 62.5 years, divided by 10. Accordingly, the five year age groups are coded as: 25-29 = -3.5, 30-34 = -3.0, …., 60-64 = 0, …, 90-94 = 3.0, 95+ = 3.5
- Age_Sq is the above age variable, squared
- Year is a dummy variable for the seven financial years
- Dis_Phase_j,k_ is the disease phase coding for the j=6 NCDs (cancer, CVD, LLK, DM, Neurological and musculoskeletal), each categorized to one of k categories (0 = nil disease; 1 = first year of diagnosis; 2 = prevalent; 3 = in financial year of death from disease)
- Dis is a binary dummy variable for having the j=6 NCDs, i.e. coded as ‘1’ if any disease phase, 0 if nil disease – a collapsed binary dummy variable of Dis_Phase_j,k_. It is only used in the disease-disease comorbidity pairs, such that there are 15 possible combinations.

And the coefficients include:

- α as the intercept, namely the expected non-NCD expenditure for the reference person (i.e. age 60-64 in financial year 2011-12 with none of the 6 (or 13) NCDs)
- ε is the normally distributed error term
- and the other coefficients accord to the given variables.

And:

- Notation for each variable for individual i in year t is suppressed to avoid clutter.

For the regression models with no comorbidity pairs, the terms $\sum_{j=1}^{6} \sum_{l=j+1}^{6} \varphi_{i,j}.{Dis}_{i}.{Dis}_{l}$ and $\sum_{j=1}^{6} \sum_{l=j+1}^{6} \omega_{i,j}.{Dis}_{i}.{Dis}_{l}.Age$ are not included.

For the 13-disease models, the upper limit of j are simply changed to 13 for the main effects (i.e. those with Dis_Phase) and the disaggregated list of 13 diseases uses, but left at 6 for the disease comorbidity pair terms (as it was not practicable or interpretable to have all 78 comorbidity pars formed by 13 diseases. “

## **Comparison of between person OLS regression to within person fixed effects regression analyses**

The main analyses in this paper used weighted ordinary least squares regression on aggregated data. The aggregation was into unique strata formed by cross-classifying five-year age groups, by financial year, by the four levels of each disease (nil diagnosis, diagnosed in that financial year, dying of that disease in that financial year, otherwise prevalent with that disease). For the disaggregated 13 disease classifications, there were 151,913 unique strata of these cross-classified categorical variables with at least one male observation, and 139,717 unique strata for females. The between person OLS regressions were run on these aggregated datasets, with weights equal to the number of observations in each strata.

The main OLS regressions give the between person differences in expenditure between strata. A limitation of this approach is residual confounding by time invariant characteristics, for example some people be higher users of health services and also (as a consequence) more likely to be diagnosed with conditions.

A fixed effects analysis can examine within individual changes in expenditure with the onset of disease phase, with all time invariant confounding dropping out. However, this gain comes at a greater level of exactitude placed on the data coding. For example, the onset of diabetes is difficult to pinpoint in time, and therefore demarcating the boundary between ‘diabetes free’ and ‘year of diagnosis with diabetes’ is somewhat arbitrary. Consequently, if costs had been slowly increasing during a pre-diabetes phase (coded as disease-free in our modelling), the fixed effects may underestimate the diagnostic year excess costs relative to a person without diabetes. Also, conceptually the changes in within person costs is not the same entity as the between person costs. For example, someone dying of diabetes probably had more severe (and costly) diabetes in the prevalent phase, which may see the cost of diabetes in the last year of life be less according to a within person compared to a between person conceptualization.

Nevertheless, a fixed effects analyses does offer a robustness check on our main between person OLS analyses. Supplementary Table 7 shows the fixed effects analyses, compared to the main between person OLS analyses reported in the main paper (but with coefficients in NZ$ 2011). Ignoring the age interactions and focusing on disease terms give results for 60-64 year olds, as age was centred on 62.5 years. The obvious anomaly is the large negative cost in the within person fixed effects analyses for year of death from diabetes, for both males (-$15,796) and females (-$12,406). The reasons for this were: a) diabetics who are destined to die of diabetes, have steadily increasing costs in the years leading up to death – not just the year of death; and b) the date of death can occur anywhere in the last financial year the person died in, an average of six months person time. For example, for females who died of diabetes their average total costs in the year they died was NZ$20,197, and their average total costs in the financial year immediately preceding the death year was $25,287 (or a difference of -$5070). That is, they cost less in the last year of life. The coefficient we see in Supplementary Table 7 is more negative again (-$12,406) due to the contribution of comorbidities in the regression modelling. Thus, for diseases with increasing costs leading up to the year of death, within our 7-year window of analyses, will see the within person fixed effects and between person OLS giving appropriately different results – even to the point of negative costs in the fixed effects analyses due to the average of six months of follow-up time.

(Of note, we could have doubled the last year of life costs for all observations, to return a cost that is – on average – per person-year. We chose not to do that, as one motivation for our analyses was to generate disease off-set costs for a macrosimulation model where – indeed – the average duration of life in the last annual cycle is six months.)

Putting the diabetes death costs aside, Supplementary Figure 2 plots the OLS between person against the fixed effects within person results. Overlaid are regression slopes (forced to go through origin) and correlation coefficients. Agreement is reasonable. For example, the year of diagnosis costs have an approximately 1:1 relationship (regression coefficients 0.91 and 0.99 for males and females, respectively) and reasonable correlations (0.72 and 0.37 – noting only six diseases plotted in each instance).

In summary, due to conceptual and data reasons, we do not expect the between person OLS and within person fixed effects analyses to be identical – but similar. This is what we found.
