# Supplementary material for: Health system costs for individual and comorbid noncommunicable diseases: An analysis of publicly funded health events from New Zealand
Source: PLoS Med. 2019 Jan 8;16(1):e1002716. doi: 10.1371/journal.pmed.1002716 (PMC6324792; doi:10.1371/journal.pmed.1002716)
Supplement: S1 Table — (DOCX) [file pmed.1002716.s003.docx]

| **Major Grouping** | **Detailed Disease Grouping** | **Sub-Diseases or Conditions** | **ICD10 Mortality Codes** | **ICD10 Case Definition Codes (if different from mort.)** | **Source Data for definition** | **Case Definition Rules** |
| --- | --- | --- | --- | --- | --- | --- |
| Cancer | Overall |  | C00-C96 |  | Cancer registry | First diagnostic date of any of these cancers |
|  | Lung |  | C33-C34 |  | Cancer registry |  |
|  | Colorectal |  | C18-C20 |  | Cancer registry |  |
|  | Breast |  | C50 |  | Cancer registry |  |
|  | Prostate |  | C61 |  | Cancer registry |  |
|  | Other Cancers |  |  |  | Cancer registry |  |
| Diabetes | Type II DM |  |  |  | NMDS primary diagnosis or VDR | Omit Type I Diabetes |
| Cardiovascular disease | Any CVD |  | I00-I72.4, I72.8-I72.9, I73.8-I73.9, I79.0, I98.0-I98.1 |  | NMDS primary diagnosis |  |
|  | IHD |  | I20-I25 |  | NMDS primary diagnosis |  |
|  | IHD (contd) |  |  |  | PHARMS dispensing (for Angina) | Angina definition : from PHARMS where chemical_id in (1577 [Glyceryl trinitrate], 2377 [Isosorbide dinitrate], 2836 [Isosorbide mononitrate], 1272 [Nicorandil], 1949 [Perhexiline maleate]) but not chemical_id eq 1577 and presentation_id eq 26 [Oint 0.2%]), dispensed on two different days within a year. |
|  | IHD (contd) |  |  |  | NMDS procedures | Any of : 3530400, 3530500, 3531000, 3531001, 3531002, 3849700, 3849701, 3849702, 3849703, 3849704, 3849705, 3849706, 3849707, 3850000, 3850001, 3850002, 3850003, 3850004, 3850300, 3850301, 3850302, 3850303, 3850304, 3863700, 9020100, 9020101, 9020102, 9020103 |
|  | Stroke |  | G45-G46,I60-I66,I67.9 |  | NMDS primary diagnosis |  |
| Chronic lung, liver and kidney | COPD |  | J40-J44 |  |  |  |
|  | Chronic liver disease |  | K70-K71.1, K71.3-K76.9 |  |  |  |
|  | Chronic kidney disease |  | I12, I13.1, N03-N07, N11-N13, Q60-Q61.9 |  |  |  |
|  |  |  |  |  |  |  |
| Neurological Conditions | Any |  | F00-F01, F03-F06, F51.0, F70-F79, G08, G10-G13, G20-G44, G47.0-G47.2, G47.8-G73.6, G90-G93, G95-G99, H81.4 |  |  |  |
|  |  | Epilepsy |  | G40-G41 | NMDS diagnosis (primary or other) | Require 2 or more events with ICD codes |
|  |  | Epilepsy (contd) |  |  | PHARMS dispensing | Any of : 1004 [Vigabatrin], 1481 [Ethosuximide], 1956 [Phenobarbitone sodium], 1978 [Phenytoin sodium], 2041 [Primidone], 3354 [Phenobarbitone] |
|  |  | Epilepsy (contd) |  | G40-G41 | NMDS diagnosis and PHARMS dispensing | One diagnosis event and one or more dispensing of : 1308 [Clobazam], 1002 [Lamotrigine], 1062 [Gabapentin], 1133 [Topiramate], 2059 [Paraldehyde], 2166 [Sodium valproate], 1316 [Clonazepam], 1217 [Carbamazepine], 1397 [Diazepam] |
|  |  | Dementia |  | F00, F03, G30 | NMDS diagnosis (primary or other) | Note G31 excluded from this definition but included in major grouping |
|  |  | Dementia (contd) |  |  | PHARMS dispensing | Any of : 3923 [Donepezil hydrochloride], 3750 [Rivastigmine] |
|  |  | Parkinson's disease |  | G20 | NMDS diagnosis (primary or other) | May be additional PHARMS not included |
|  |  | Multiple sclerosis |  | G35 | NMDS diagnosis (primary or other) | May be additional PHARMS not included |
|  |  | Motor neuron disease |  | G12.2 | NMDS diagnosis (primary or other) | May be additional PHARMS not included |
|  |  | Muscular dystrophy |  | G70-G73 | NMDS diagnosis (primary or other) | May be additional PHARMS not included |
|  |  | Migraine |  | G43 | NMDS diagnosis (primary or other) |  |
|  |  | Migraine (contd) |  |  | PHARMS dispensing | Any of : 1214 [Dihydroergotamine mesylate], 1458 [Ergotamine tartrate], 1459 [Ergotamine tartrate with cyclizine], 1460 [Ergotamine tartrate with diphenhydramine], 1462 [Ergotamine tartrate with caffeine], 1814 [Metoclopramide hydrochloride], 1815 [Metoclopramide hydrochloride with paracetamol], 2000 [Pizotifen], 2800 [Sumatriptan], 3876 [Rizatriptan] |
|  |  | Intellectual impairment |  | F70-F79 | NMDS diagnosis (primary or other) |  |
|  |  | Intellectual impairment (contd) |  |  | NMDS Health Specialty Code | Health Specialty event D60-D74 (Intellectual disability) |
|  |  | Intellectual impairment (contd) |  |  | PRIMHD Team Type | Contact with team type 12 (Intellectual disability dual diagnosis team) |
|  |  | Sleep disorders |  | F51.0, G47.0 | NMDS diagnosis (primary or other) |  |
|  |  | Other neurological conditions |  | F04-F06, G08, G10-G12.1, G12.8-G13.0, G21-G26, G32, G36-G37, G44, G47.1-G47.2, G47.8-G64.9, G90-G93, G95-G99 | NMDS diagnosis (primary or other) |  |
|  |  |  |  |  |  |  |
| Musculoskeletal Disorders |  |  | M02, M05-M08, M10-M35, M40-M46.1, M46.4, M47-M48, M50-M54, M60.1-M62.99, M65.2-M67.99, M70, M71.2-M72.49, M72.8-M72.99, M75-M81, M83, M84.4-M85.99, M87-M89, M91-M99 |  |  |  |
|  |  | Osteoarthritis (OA) |  | M15-M19, M47 | NMDS diagnosis (primary or other) | Note M47 included (some other definitions exclude this) |
|  |  | Gout |  | M10, M11 | NMDS diagnosis (primary or other) | Note M11 included (some other definitions exclude this) |
|  |  | Gout (contd) |  |  | PHARMS dispensing | One or more dispensing of : 1341 [Colchicine] |
|  |  | Gout (contd) |  |  | PHARMS and (Cancer registry or NMDS) | One or more dispensing of 1026 [Allopurinol] as long as there is no C81-C96 recorded within 24 months |
|  |  | Rheumatoid arthritis (RA) |  | M05-M06 | NMDS diagnosis (primary or other) |  |
|  |  | SLE |  | M32 | NMDS diagnosis (primary or other) |  |
|  |  | Other rheumatological conditions |  | M02, M07-M08, M12-M13, M30-M31, M33-M35, M45, M60.1-M60.99 | NMDS diagnosis (primary or other) |  |
|  |  | Spinal disorders |  | M48-M48.2, M48.8-M48.9, M50-M54 | NMDS diagnosis (primary or other) |  |
|  |  | Chronic MSK pain syndromes |  | M70.8-M70.9, M75-M77.9, M79.7 | NMDS diagnosis (primary or other) |  |
|  |  | Other non-arthritis MSK conditions |  | M14, M20-M25, M40-M43, M46-M46.1, M46.4-M46.49, M48.3-M48.5, M61-M62, M65.2-M67.99, M70-M70.7, M71.2-M72.49, M72.8-M72.99, M79-M79.6, M79.8-M81.9, M83, M84.4-M85.9, M87-M89.9, M91-M99.9 | NMDS diagnosis (primary or other) |  |

NMDS = national minimum dataset; VDR = virtual diabetes register; PRIMHD = New Zealand Ministry of Health single national mental health and addiction information collection of service activity and outcomes data for health consumers.
