# Supplementary material for: Health system costs for individual and comorbid noncommunicable diseases: An analysis of publicly funded health events from New Zealand
Source: PLoS Med. 2019 Jan 8;16(1):e1002716. doi: 10.1371/journal.pmed.1002716 (PMC6324792; doi:10.1371/journal.pmed.1002716)
Supplement: S2 Table — (DOCX) [file pmed.1002716.s004.docx]

|  | **Inpatient** | **Outpatient** | **Pharmaceutical** | **Laboratory** | **Primary care** | **Total** |
| --- | --- | --- | --- | --- | --- | --- |
| ***Disease separately*** |  |  |  |  |  |  |
| Cancer | $3,846 | $1,891 | $1,255 | $146 | $198 | $7,338 |
| Lung cancer | $256 | $116 | $47 | $5 | $6 | $431 |
| Colorectal cancer | $699 | $246 | $120 | $18 | $25 | $1,107 |
| Breast cancer | $529 | $439 | $315 | $28 | $46 | $1,358 |
| Prostate cancer | $586 | $286 | $185 | $32 | $45 | $1,134 |
| Other cancer | $1,776 | $804 | $589 | $63 | $76 | $3,308 |
| Cardiovascular disease (CVD) | $12,993 | $4,405 | $3,831 | $546 | $908 | $22,695 |
| IHD | $4,224 | $1,200 | $1,148 | $144 | $260 | $6,979 |
| Stroke | $1,718 | $452 | $399 | $54 | $98 | $2,723 |
| Other CVD | $3,752 | $1,146 | $842 | $127 | $188 | $6,058 |
| Type 2 diabetes mellitus (DM) | $3,299 | $1,609 | $1,441 | $221 | $362 | $6,935 |
| Chronic lung/liver/kidney (LLK) disease | $19,180 | $7,258 | $5,830 | $785 | $1,307 | $34,378 |
| Chronic lung disease | $1,833 | $493 | $493 | $45 | $85 | $2,951 |
| Chronic kidney disease | $991 | $797 | $265 | $36 | $44 | $2,133 |
| Chronic liver disease | $668 | $226 | $207 | $22 | $27 | $1,151 |
| Neurological (Neuro) | $7,719 | $2,946 | $2,529 | $344 | $557 | $14,103 |
| Musculoskeletal (MS) | $7,970 | $2,795 | $2,337 | $339 | $593 | $14,040 |
| ***Disease comorbidity combinations*** |  |  |  |  |  |  |
| Cancer and CVD | $1,302 | $451 | $317 | $38 | $52 | $2,161 |
| Cancer and DM | $644 | $291 | $200 | $24 | $33 | $1,193 |
| Cancer and LLK | $736 | $266 | $170 | $16 | $21 | $1,209 |
| Cancer and Neuro | $1,939 | $878 | $594 | $51 | $63 | $3,526 |
| Cancer and MS | $1,577 | $635 | $436 | $47 | $62 | $2,760 |
| CVD and DM | $1,683 | $689 | $491 | $61 | $93 | $3,017 |
| CVD and LLK | $1,674 | $689 | $397 | $41 | $62 | $2,864 |
| CVD and Neuro | $3,289 | $1,068 | $788 | $92 | $145 | $5,385 |
| CVD and MS | $3,730 | $1,169 | $904 | $116 | $184 | $6,106 |
| DM and LLK | $844 | $462 | $244 | $27 | $37 | $1,614 |
| DM and Neuro | $1,687 | $864 | $554 | $66 | $97 | $3,270 |
| DM and MS | $1,827 | $859 | $601 | $82 | $127 | $3,497 |
| LLK and Neuro | $1,765 | $742 | $447 | $44 | $60 | $3,060 |
| LLK and MS | $1,856 | $813 | $478 | $49 | $70 | $3,270 |
| Neuro and MS | $4,071 | $1,461 | $1,114 | $128 | $198 | $6,978 |
