# Supplementary material for: Health system costs for individual and comorbid noncommunicable diseases: An analysis of publicly funded health events from New Zealand
Source: PLoS Med. 2019 Jan 8;16(1):e1002716. doi: 10.1371/journal.pmed.1002716 (PMC6324792; doi:10.1371/journal.pmed.1002716)
Supplement: S3 Table — (DOCX) [file pmed.1002716.s005.docx]

S3 Table. Additional regression coefficients (and s.e.; NZ$ for 2011) for the age-by-disease, age squared-by-disease and age-by-disease-by-disease interactions.

|  |  | **Males** |  |  |  | **Females** |  |  |  |
| --- | --- | --- | --- | --- | --- | --- | --- | --- | --- |
|  |  | **6 diseases** |  | **13 diseases** |  | **6 diseases** |  | **13 diseases** |  |
| **Variable** |  | **Base model** | **+ disease interactions** | **Base model** | **+ disease interactions** | **Base model** | **+ disease interactions** | **Base model** | **+ disease interactions** |
| Intercept |  | 475(37) | 848(36) | 505(32) | 814(32) | 601(31) | 876(30) | 613(27) | 852(27) |
| Age ‡ |  | 215(17) | 273(19) | 238(14) | 271(16) | 229(12) | 262(13) | 239(11) | 259(12) |
| Age squared ‡ |  | 67(6) | 53(6) | 71(5) | 56(5) | 103(5) | 93(5) | 105(4) | 94(4) |
| DISEASE-PHASE MAIN EFFECTS | | | | | | | | | |
| Disease main effects – first year of diagnosis | | | | | | | | | |
| Cancer |  | 13007(207) | 11777(204) |  |  | 16309(193) | 15097(188) |  |  |
|  | Lung |  |  | 17258(707) | 15960(699) |  |  | 17781(626) | 16155(618) |
|  | Colorectal |  |  | 22994(472) | 21970(467) |  |  | 22470(473) | 21308(467) |
|  | Breast |  |  |  |  |  |  | 13515(289) | 12618(286) |
|  | Prostate |  |  | 4527(278) | 3884(277) |  |  |  |  |
|  | Other |  |  | 17137(268) | 15926(270) |  |  | 15680(251) | 14496(250) |
| CVD |  | 12582(160) | 11642(156) |  |  | 10002(164) | 8911(160) |  |  |
|  | IHD |  |  | 11918(186) | 11309(185) |  |  | 8665(206) | 7835(205) |
|  | Stroke |  |  | 8491(276) | 7767(273) |  |  | 9598(275) | 8752(273) |
|  | Other CVD |  |  | 13865(198) | 13302(197) |  |  | 11044(211) | 10365(209) |
| DM |  | 741(191) | 108(183) | 730(165) | 120(165) | 436(167) | -14(160) | 432(150) | -7(149) |
| Chronic LLK |  | 12017(255) | 8820(257) |  |  | 11134(237) | 8780(236) |  |  |
|  | Chronic lung |  |  | 11499(324) | 8912(328) |  |  | 9835(292) | 7905(294) |
|  | CKD |  |  | 10741(363) | 8452(364) |  |  | 11254(366) | 9466(366) |
|  | CLD |  |  | 13640(447) | 11153(447) |  |  | 12536(465) | 10647(463) |
| Neurological |  | 8551(136) | 6710(136) | 8089(118) | 6370(123) | 5004(96) | 4089(94) | 4866(86) | 3995(87) |
| Musculoskeletal |  | 5480(129) | 4871(125) | 5370(112) | 4829(113) | 7776(127) | 7121(124) | 7684(114) | 7063(115) |
| Disease main effects – last year of life if dying of disease | | | | | | | | | |
| Cancer |  | 15380(316) | 12149(319) |  |  | 16422(285) | 13628(284) |  |  |
|  | Lung |  |  | 11452(589) | 9153(587) |  |  | 12577(551) | 10077(549) |
|  | Colorectal |  |  | 15618(719) | 13196(713) |  |  | 16145(710) | 13714(703) |
|  | Breast |  |  |  |  |  |  | 15797(623) | 13234(618) |
|  | Prostate |  |  | 12632(1073) | 9559(1061) |  |  |  |  |
|  | Other |  |  | 16995(370) | 14235(378) |  |  | 17809(368) | 15123(371) |
| CVD |  | 13573(486) | 11614(462) |  |  | 14766(522) | 12759(498) |  |  |
|  | IHD |  |  | 9545(549) | 8205(542) |  |  | 11316(829) | 9497(817) |
|  | Stroke |  |  | 8526(900) | 7464(886) |  |  | 11030(766) | 10128(755) |
|  | Other CVD |  |  | 18641(927) | 17502(913) |  |  | 18310(958) | 17058(944) |
| DM |  | 23848(1053) | 19688(999) | 22639(912) | 18976(902) | 26842(1089) | 23364(1036) | 25783(976) | 22870(964) |
| Chronic LLK |  | 15188(814) | 11400(774) |  |  | 15481(735) | 12518(702) |  |  |
|  | Chronic lung |  |  | 11500(924) | 8797(912) |  |  | 11840(770) | 9562(761) |
|  | CKD |  |  | 35643(2606) | 31066(2567) |  |  | 46503(2862) | 43210(2818) |
|  | CLD |  |  | 12116(1688) | 9469(1662) |  |  | 14167(2090) | 12351(2057) |
| Neurological |  | 6401(953) | 5038(901) | 6394(825) | 5103(813) | 5539(825) | 4783(782) | 5569(739) | 4846(728) |
| Musculosk. |  | 26896(3097) | 24708(2923) | 26081(2681) | 24308(2638) | 21449(1902) | 19778(1803) | 20739(1705) | 19286(1679) |
| Disease main effects – prevalent years of diagnosis | | | | | | | | | |
| Cancer |  | 2861(87) | 1674(100) |  |  | 2872(71) | 1793(78) |  |  |
|  | Lung |  |  | 5262(635) | 3847(630) |  |  | 5924(527) | 4209(523) |
|  | Colorectal |  |  | 4407(223) | 3350(225) |  |  | 3359(211) | 2231(211) |
|  | Breast |  |  |  |  |  |  | 2172(87) | 1261(92) |
|  | Prostate |  |  | 1187(116) | 494(121) |  |  |  |  |
|  | Other |  |  | 3628(112) | 2549(122) |  |  | 2798(99) | 1748(105) |
| CVD |  | 2428(58) | 1393(68) |  |  | 2407(59) | 1161(71) |  |  |
|  | IHD |  |  | 1603(64) | 990(68) |  |  | 1837(72) | 931(78) |
|  | Stroke |  |  | 1300(112) | 529(115) |  |  | 1299(110) | 421(114) |
|  | Other CVD |  |  | 2285(75) | 1732(77) |  |  | 2352(79) | 1663(83) |
| DM |  | 1877(63) | 849(73) | 1822(54) | 852(66) | 1704(55) | 977(63) | 1667(50) | 983(59) |
| Chronic LLK |  | 4914(102) | 1455(133) |  |  | 4251(86) | 1785(110) |  |  |
|  | Chronic lung |  |  | 3962(133) | 1115(154) |  |  | 3666(110) | 1490(128) |
|  | CKD |  |  | 7015(150) | 4439(165) |  |  | 5558(129) | 3827(140) |
|  | CLD |  |  | 2747(168) | 361(180) |  |  | 2558(166) | 902(174) |
| Neurological |  | 2224(59) | 502(72) | 2144(51) | 519(65) | 1439(40) | 532(45) | 1405(36) | 543(42) |
| Musculoskeletal |  | 1265(45) | 601(52) | 1198(39) | 611(47) | 1814(45) | 1115(52) | 1767(41) | 1111(48) |
| Disease comorbidity interactions | | | | | | | | | |
| Cancer & CVD |  |  | -340(191) |  | -669(172) |  | -35(183) |  | -171(170) |
| Cancer & DM |  |  | 29(216) |  | -124(195) |  | 22(165) |  | 68(153) |
| Cancer & LLK |  |  | 383(253) |  | -345(229) |  | 506(208) |  | 246(194) |
| Cancer and Neuro |  |  | 4371(150) |  | 3854(136) |  | 3368(108) |  | 3219(101) |
| Cancer and MS |  |  | 1446(145) |  | 1507(131) |  | 1012(129) |  | 1111(120) |
| CVD and DM |  |  | 1486(122) |  | 1294(110) |  | 1419(122) |  | 1254(114) |
| CVD and LLK |  |  | 3455(165) |  | 2835(150) |  | 3397(154) |  | 2947(145) |
| CVD and Neuro |  |  | 1384(107) |  | 1291(97) |  | 1179(93) |  | 1002(87) |
| CVD and MS |  |  | 904(93) |  | 596(82) |  | 901(100) |  | 654(93) |
| DM and LLK |  |  | 1857(177) |  | 1605(160) |  | 1257(150) |  | 964(140) |
| DM and Neuro |  |  | 2336(117) |  | 2354(106) |  | 1155(88) |  | 1168(82) |
| DM and MS |  |  | 554(97) |  | 587(88) |  | 408(94) |  | 436(87) |
| LLK and Neuro |  |  | 2387(150) |  | 2171(135) |  | 1435(115) |  | 1206(107) |
| LLK and MS |  |  | 2393(141) |  | 1942(127) |  | 1576(121) |  | 1359(112) |
| Neuro and MS |  |  | 1099(86) |  | 1129(78) |  | 1020(66) |  | 1045(62) |
| AGE-BY-DISEASE COEFFICIENTS | | | | | | | | | |
| Age-by-disease coefficients – first year of diagnosis | | | | | | | | | |
| Cancer |  | -1836(129) | -1182(129) |  |  | -1378(98) | -906(98) |  |  |
|  | Lung |  |  | -2592(585) | -1614(577) |  |  | -2944(476) | -2241(469) |
|  | Colorectal |  |  | -1346(319) | -754(316) |  |  | -1537(276) | -949(273) |
|  | Breast |  |  |  |  |  |  | -2363(155) | -1907(155) |
|  | Prostate |  |  | 335(330) | 610(326) |  |  |  |  |
|  | Other |  |  | -2364(140) | -1716(142) |  |  | -1470(122) | -984(124) |
| CVD |  | -633(90) | -316(89) |  |  | -922(85) | -503(85) |  |  |
|  | IHD |  |  | -1134(115) | -852(114) |  |  | -1000(139) | -583(139) |
|  | Stroke |  |  | -1514(169) | -1132(168) |  |  | -1823(158) | -1405(157) |
|  | Other CVD |  |  | 583(103) | 853(103) |  |  | -297(99) | 46(99) |
| DM |  | 199(137) | 230(132) | 186(118) | 225(119) | -64(108) | -57(105) | -61(97) | -51(97) |
| Chronic LLK |  | 50(120) | 837(126) |  |  | 12(100) | 409(104) |  |  |
|  | Chronic lung |  |  | -635(184) | 205(187) |  |  | -437(153) | 60(155) |
|  | CKD |  |  | 1249(175) | 1925(178) |  |  | 867(177) | 1230(178) |
|  | CLD |  |  | -867(245) | -5(246) |  |  | -713(234) | -231(233) |
| Neurological |  | 822(59) | 821(64) | 766(51) | 784(58) | 789(39) | 715(40) | 750(35) | 685(38) |
| Musculoskeletal |  | 1174(72) | 1142(72) | 1136(62) | 1125(65) | 615(57) | 734(57) | 591(51) | 723(53) |
| Age-by-disease coefficients – last year of life if dying of disease | | | | | | | | | |
| Cancer |  | -4543(222) | -2835(223) |  |  | -4411(172) | -3160(174) |  |  |
|  | Lung |  |  | -3793(573) | -2392(567) |  |  | -3909(469) | -2754(465) |
|  | Colorectal |  |  | -3387(511) | -1982(506) |  |  | -3820(467) | -2667(462) |
|  | Breast |  |  |  |  |  |  | -4715(326) | -3513(325) |
|  | Prostate |  |  | -4905(1277) | -3023(1258) |  |  |  |  |
|  | Other |  |  | -4671(231) | -3059(237) |  |  | -4590(213) | -3298(217) |
| CVD |  | -3614(381) | -2742(361) |  |  | -4497(394) | -3689(375) |  |  |
|  | IHD |  |  | -3351(486) | -2619(479) |  |  | -3769(752) | -2732(741) |
|  | Stroke |  |  | -3806(634) | -3173(624) |  |  | -4674(512) | -4298(504) |
|  | Other CVD |  |  | -3045(615) | -2296(605) |  |  | -4049(649) | -3242(639) |
| DM |  | -11502(979) | -9696(927) | -11112(848) | -9567(837) | -15450(969) | -14199(920) | -15290(868) | -14247(857) |
| Chronic LLK |  | -6017(705) | -4500(669) |  |  | -6652(614) | -5742(584) |  |  |
|  | Chronic lung |  |  | -3099(1010) | -1848(995) |  |  | -3687(820) | -2954(808) |
|  | CKD |  |  | -13469(1363) | -11492(1343) |  |  | -17619(1314) | -16133(1294) |
|  | CLD |  |  | -2955(1123) | -2056(1106) |  |  | -2069(1115) | -1506(1098) |
| Neurological |  | -1450(553) | -1410(523) | -1388(479) | -1342(472) | -1636(568) | -1684(538) | -1577(509) | -1629(501) |
| Musculosk. |  | -3655(1788) | -2950(1688) | -3252(1548) | -2704(1523) | -4705(1101) | -3992(1043) | -4484(987) | -3846(971) |
| Age-by-disease coefficients – prevalent years of diagnosis | | | | | | | | | |
| Cancer |  | -448(51) | 158(64) |  |  | -629(39) | -175(49) |  |  |
|  | Lung |  |  | -1079(507) | -160(501) |  |  | -1549(383) | -841(379) |
|  | Colorectal |  |  | -1226(164) | -635(165) |  |  | -1083(142) | -533(142) |
|  | Breast |  |  |  |  |  |  | -1421(56) | -969(61) |
|  | Prostate |  |  | -126(161) | 159(161) |  |  |  |  |
|  | Other |  |  | -310(57) | 270(65) |  |  | -185(49) | 275(56) |
| CVD |  | -27(34) | 314(43) |  |  | -159(31) | 284(40) |  |  |
|  | IHD |  |  | -167(46) | 124(49) |  |  | -187(54) | 251(57) |
|  | Stroke |  |  | -227(72) | 183(74) |  |  | -330(63) | 71(66) |
|  | Other CVD |  |  | -92(38) | 189(41) |  |  | -224(36) | 132(39) |
| DM |  | -143(37) | -52(51) | -142(32) | -51(46) | -134(29) | -112(39) | -130(26) | -107(36) |
| Chronic LLK |  | -451(48) | 406(74) |  |  | -104(36) | 271(55) |  |  |
|  | Chronic lung |  |  | -207(68) | 647(83) |  |  | -135(54) | 346(66) |
|  | CKD |  |  | -797(76) | 16(87) |  |  | 48(66) | 386(73) |
|  | CLD |  |  | -433(102) | 323(109) |  |  | -399(85) | 5(90) |
| Neurological |  | 96(30) | 80(43) | 98(26) | 101(39) | 72(19) | -3(26) | 72(17) | 5(24) |
| Musculoskeletal |  | 274(26) | 248(35) | 256(22) | 253(31) | 120(20) | 236(27) | 110(18) | 240(25) |
| Age-by-disease-by-disease coefficients | | | | | | | | | |
| Cancer & CVD |  |  | 77(131) |  | 222(117) |  | -130(115) |  | -140(106) |
| Cancer & DM |  |  | -172(159) |  | -94(143) |  | 12(120) |  | 16(112) |
| Cancer & LLK |  |  | -276(166) |  | -100(150) |  | -555(135) |  | -514(126) |
| Cancer and Neuro |  |  | -1762(100) |  | -1671(91) |  | -1166(72) |  | -1152(67) |
| Cancer and MS |  |  | -775(102) |  | -757(92) |  | -496(85) |  | -531(79) |
| CVD and DM |  |  | -512(96) |  | -433(87) |  | -440(83) |  | -400(78) |
| CVD and LLK |  |  | -1876(114) |  | -1741(103) |  | -1613(97) |  | -1556(91) |
| CVD and Neuro |  |  | -626(74) |  | -603(67) |  | -432(57) |  | -376(52) |
| CVD and MS |  |  | -257(68) |  | -244(60) |  | -377(61) |  | -349(56) |
| DM and LLK |  |  | -860(126) |  | -824(114) |  | -402(98) |  | -337(91) |
| DM and Neuro |  |  | -431(82) |  | -441(74) |  | -49(56) |  | -56(52) |
| DM and MS |  |  | -155(74) |  | -163(66) |  | -170(63) |  | -175(59) |
| LLK and Neuro |  |  | -978(93) |  | -925(84) |  | -183(60) |  | -181(55) |
| LLK and MS |  |  | -849(91) |  | -713(82) |  | -599(68) |  | -585(63) |
| Neuro and MS |  |  | -66(50) |  | -70(45) |  | -145(36) |  | -146(34) |
| AGE-SQUARED-BY-DISEASE COEFFICIENTS | | | | | | | | | |
| Age-squared-by-disease coefficients – first year of diagnosis | | | | | | | | | |
| Cancer |  | 209(72) | 182(69) |  |  | -342(56) | -330(54) |  |  |
|  | Lung |  |  | -846(320) | -972(315) |  |  | -718(278) | -661(273) |
|  | Colorectal |  |  | -174(176) | -189(173) |  |  | -186(150) | -209(147) |
|  | Breast |  |  |  |  |  |  | 12(93) | -6(92) |
|  | Prostate |  |  | -202(185) | -152(183) |  |  |  |  |
|  | Other |  |  | -564(79) | -569(78) |  |  | -524(67) | -508(66) |
| CVD |  | -356(52) | -327(49) |  |  | -147(46) | -136(43) |  |  |
|  | IHD |  |  | -487(70) | -483(69) |  |  | -188(71) | -204(70) |
|  | Stroke |  |  | 257(92) | 248(90) |  |  | 144(78) | 129(76) |
|  | Other CVD |  |  | -245(58) | -236(57) |  |  | -160(54) | -171(53) |
| DM |  | 160(70) | 144(66) | 152(60) | 138(60) | 233(50) | 223(48) | 231(45) | 223(44) |
| Chronic LLK |  | -335(67) | -38(65) |  |  | -410(52) | -217(50) |  |  |
|  | Chronic lung |  |  | -353(96) | -108(95) |  |  | -232(81) | -75(80) |
|  | CKD |  |  | 14(94) | 245(93) |  |  | -264(80) | -109(79) |
|  | CLD |  |  | -11(131) | 174(129) |  |  | -184(124) | -40(123) |
| Neurological |  | -393(30) | -247(29) | -358(26) | -218(26) | -96(18) | -49(18) | -95(16) | -49(17) |
| Musculoskeletal |  | 17(34) | 34(33) | 15(29) | 34(29) | -193(29) | -175(28) | -195(26) | -174(26) |
| Age-squared-by-disease coefficients – last year of life if dying of disease | | | | | | | | | |
| Cancer |  | 61(108) | -28(102) |  |  | -192(85) | -229(81) |  |  |
|  | Lung |  |  | -21(286) | -123(281) |  |  | -10(242) | -19(238) |
|  | Colorectal |  |  | -273(245) | -357(241) |  |  | -153(206) | -186(203) |
|  | Breast |  |  |  |  |  |  | -221(182) | -256(180) |
|  | Prostate |  |  | 347(424) | 190(417) |  |  |  |  |
|  | Other |  |  | -26(120) | -133(119) |  |  | -348(103) | -397(101) |
| CVD |  | -37(148) | -52(139) |  |  | 67(126) | 65(120) |  |  |
|  | IHD |  |  | 217(179) | 170(176) |  |  | 117(208) | 11(205) |
|  | Stroke |  |  | 502(261) | 454(257) |  |  | 441(184) | 453(181) |
|  | Other CVD |  |  | -803(263) | -886(258) |  |  | -488(233) | -588(229) |
| DM |  | 1105(444) | 925(420) | 1142(385) | 990(379) | 2210(369) | 2125(349) | 2274(331) | 2194(325) |
| Chronic LLK |  | 752(280) | 853(264) |  |  | 730(240) | 799(228) |  |  |
|  | Chronic lung |  |  | 58(348) | 143(343) |  |  | 37(291) | 139(287) |
|  | CKD |  |  | 1749(698) | 1866(687) |  |  | 739(672) | 707(661) |
|  | CLD |  |  | -182(693) | -33(682) |  |  | -76(663) | -17(653) |
| Neurological |  | -162(218) | -75(205) | -157(188) | -70(185) | -126(176) | -93(167) | -139(158) | -103(156) |
| Musculosk. |  | -1618(812) | -1624(766) | -1619(703) | -1614(691) | -661(444) | -729(420) | -665(398) | -722(392) |
| Age-squared-by-disease coefficients – prevalent years of diagnosis | | | | | | | | | |
| Cancer |  | -39(28) | -47(28) |  |  | -28(22) | -29(22) |  |  |
|  | Lung |  |  | -265(271) | -337(266) |  |  | -101(240) | -40(237) |
|  | Colorectal |  |  | -42(84) | -52(83) |  |  | 35(69) | 29(68) |
|  | Breast |  |  |  |  |  |  | 351(33) | 342(34) |
|  | Prostate |  |  | 5(70) | 58(70) |  |  |  |  |
|  | Other |  |  | -148(32) | -150(32) |  |  | -123(27) | -117(27) |
| CVD |  | -40(19) | -1(18) |  |  | -63(16) | -34(15) |  |  |
|  | IHD |  |  | 13(26) | 14(26) |  |  | -55(25) | -64(24) |
|  | Stroke |  |  | -44(37) | -55(37) |  |  | -7(30) | -7(30) |
|  | Other CVD |  |  | -42(21) | -39(21) |  |  | -52(19) | -64(19) |
| DM |  | -60(23) | -35(23) | -54(20) | -33(21) | -58(16) | -50(17) | -55(15) | -50(16) |
| Chronic LLK |  | -203(27) | 110(28) |  |  | -296(18) | -93(20) |  |  |
|  | Chronic lung |  |  | -226(36) | 68(37) |  |  | -229(28) | -25(29) |
|  | CKD |  |  | -357(39) | -124(40) |  |  | -398(27) | -260(28) |
|  | CLD |  |  | -34(53) | 143(52) |  |  | -133(43) | -16(43) |
| Neurological |  | -110(14) | 10(15) | -101(12) | 17(14) | -44(9) | -4(9) | -41(8) | -1(8) |
| Musculoskeletal |  | 7(12) | 31(13) | 8(11) | 33(12) | -60(10) | -38(11) | -59(9) | -35(10) |

Year was centred on 2011. Thus the coefficients in this table are in NZ$2011.

‡ Age was centred on 62.5 years, and divided by 10. Accordingly, someone in the 80-84 year age group had an age value of 2, and an age-squared value of 4.
