# Supplementary material for: Health system costs for individual and comorbid noncommunicable diseases: An analysis of publicly funded health events from New Zealand
Source: PLoS Med. 2019 Jan 8;16(1):e1002716. doi: 10.1371/journal.pmed.1002716 (PMC6324792; doi:10.1371/journal.pmed.1002716)
Supplement: S4 Table — NCD, noncommunicable disease; OLS, ordinary least squares. (DOCX) [file pmed.1002716.s006.docx]

|  |  | **Males** |  |  |  | **Females** |  |  |  |
| --- | --- | --- | --- | --- | --- | --- | --- | --- | --- |
|  |  | **6 diseases** |  | **13 diseases** |  | **6 diseases** |  | **13 diseases** |  |
| **Variable** |  | **Base model, scaled** | **+ disease interactions^‡^** | **Base model, scaled** | **+ disease interactions^‡^** | **Base model, scaled** | **+ disease interactions^‡^** | **Base model, scaled** | **+ disease interactions^‡^** |
| DISEASE-PHASE MAIN EFFECTS | | | | | | | | | |
| Disease main effects – first year of diagnosis | | | | | | | | | |
| Cancer |  | 8691 | 10093 |  |  | 9649 | 11361 |  |  |
|  | Lung |  |  | 10320 | 11708 |  |  | 11163 | 13035 |
|  | Colorectal |  |  | 13204 | 16395 |  |  | 13211 | 16095 |
|  | Breast |  |  |  |  |  |  | 9268 | 11181 |
|  | Prostate |  |  | 1915 | 1899 |  |  |  |  |
|  | Other |  |  | 10411 | 12450 |  |  | 9061 | 10721 |
| CVD |  | 6817 | 8228 |  |  | 6057 | 6767 |  |  |
|  | IHD |  |  | 6716 | 8315 |  |  | 5284 | 5965 |
|  | Stroke |  |  | 6081 | 7247 |  |  | 6865 | 8061 |
|  | Other CVD |  |  | 6671 | 8309 |  |  | 6037 | 7166 |
| DM |  | 430 | 63 | 425 | 67 | 579 | 414 | 566 | 413 |
| Chronic LLK |  | 5991 | 5407 |  |  | 5586 | 5551 |  |  |
|  | Chronic lung |  |  | 6251 | 6046 |  |  | 5407 | 5528 |
|  | CKD |  |  | 4772 | 4422 |  |  | 5077 | 5333 |
|  | CLD |  |  | 7999 | 8352 |  |  | 7155 | 7884 |
| Neurologicalc |  | 3445 | 3559 | 3290 | 3401 | 1975 | 2101 | 1913 | 2066 |
| Musculoskeletal |  | 2012 | 2339 | 1984 | 2327 | 3517 | 4068 | 3449 | 4039 |
| Disease main effects – last year of life if dying of disease | | | | | | | | | |
| Cancer |  | 11958 | 11813 |  |  | 12388 | 12908 |  |  |
|  | Lung |  |  | 9167 | 9011 |  |  | 9990 | 10241 |
|  | Colorectal |  |  | 10771 | 11110 |  |  | 11678 | 12505 |
|  | Breast |  |  |  |  |  |  | 12134 | 12962 |
|  | Prostate |  |  | 11139 | 10499 |  |  |  |  |
|  | Other |  |  | 12840 | 13393 |  |  | 12969 | 13865 |
| CVD |  | 10126 | 11286 |  |  | 11871 | 13331 |  |  |
|  | IHD |  |  | 8076 | 9049 |  |  | 9347 | 9847 |
|  | Stroke |  |  | 8240 | 9576 |  |  | 10323 | 12720 |
|  | Other CVD |  |  | 11478 | 13703 |  |  | 12630 | 14892 |
| DM |  | 23340 | 26254 | 22458 | 25706 | 30135 | 35748 | 29199 | 35555 |
| Chronic LLK |  | 13872 | 14510 |  |  | 14852 | 16578 |  |  |
|  | Chronic lung |  |  | 8730 | 8597 |  |  | 9467 | 10343 |
|  | CKD |  |  | 32060 | 37960 |  |  | 40458 | 49887 |
|  | CLD |  |  | 8655 | 9022 |  |  | 9275 | 10536 |
| Neurological |  | 4397 | 5049 | 4356 | 5031 | 4225 | 5133 | 4134 | 5103 |
| Musculosk. |  | 15388 | 18421 | 14649 | 17882 | 14806 | 17443 | 14085 | 16941 |
| Disease main effects – prevalent years of diagnosis | | | | | | | | | |
| Cancer |  | 1845 | 962 |  |  | 2057 | 1439 |  |  |
|  | Lung |  |  | 3370 | 2407 |  |  | 4350 | 3890 |
|  | Colorectal |  |  | 3299 | 3026 |  |  | 2746 | 2238 |
|  | Breast |  |  |  |  |  |  | 2762 | 2519 |
|  | Prostate |  |  | 744 | 279 |  |  |  |  |
|  | Other |  |  | 2016 | 1306 |  |  | 1518 | 775 |
| CVD |  | 1274 | 665 |  |  | 1372 | 476 |  |  |
|  | IHD |  |  | 1010 | 604 |  |  | 1082 | 297 |
|  | Stroke |  |  | 827 | 95 |  |  | 964 | 216 |
|  | Other CVD |  |  | 1249 | 984 |  |  | 1394 | 955 |
| DM |  | 1048 | 613 | 1026 | 617 | 973 | 747 | 943 | 745 |
| Chronic LLK |  | 2749 | 791 |  |  | 2050 | 845 |  |  |
|  | Chronic lung |  |  | 2019 | 215 |  |  | 1819 | 662 |
|  | CKD |  |  | 3972 | 2990 |  |  | 2490 | 1925 |
|  | CLD |  |  | 1780 | 143 |  |  | 1550 | 621 |
| Neurological |  | 982 | 293 | 949 | 294 | 675 | 382 | 654 | 385 |
| Musculoskeletal |  | 466 | 216 | 446 | 221 | 821 | 489 | 797 | 486 |
| DISEASE COMORBIDITY INTERACTIONS | | | | | | | | | |
| Cancer & CVD |  |  | -330 |  | -724 |  | 116 |  | 28 |
| Cancer & DM |  |  | 208 |  | 12 |  | 3 |  | 32 |
| Cancer & LLK |  |  | 576 |  | -141 |  | 968 |  | 735 |
| Cancer and Neuro |  |  | 5071 |  | 4599 |  | 3700 |  | 3577 |
| Cancer and MS |  |  | 1886 |  | 1911 |  | 1270 |  | 1379 |
| CVD and DM |  |  | 1630 |  | 1406 |  | 1503 |  | 1340 |
| CVD and LLK |  |  | 4532 |  | 3938 |  | 4206 |  | 3818 |
| CVD and Neuro |  |  | 1680 |  | 1588 |  | 1321 |  | 1132 |
| CVD and MS |  |  | 933 |  | 696 |  | 1061 |  | 852 |
| DM and LLK |  |  | 2275 |  | 2054 |  | 1345 |  | 1063 |
| DM and Neuro |  |  | 2157 |  | 2181 |  | 889 |  | 905 |
| DM and MS |  |  | 569 |  | 602 |  | 479 |  | 505 |
| LLK and Neuro |  |  | 2786 |  | 2573 |  | 1236 |  | 1069 |
| LLK and MS |  |  | 2651 |  | 2178 |  | 1789 |  | 1617 |
| Neuro and MS |  |  | 866 |  | 892 |  | 895 |  | 914 |

‡ The disease–disease interaction models all included 15 dummy variables for each pairwise combination of the six aggregated diseases: cancer, CVD, DM, LLK, Neuro and MS. Age and age-squared interactions with disease comorbidity interactions were also included.
