# Supplementary material for: Health system costs for individual and comorbid noncommunicable diseases: An analysis of publicly funded health events from New Zealand
Source: PLoS Med. 2019 Jan 8;16(1):e1002716. doi: 10.1371/journal.pmed.1002716 (PMC6324792; doi:10.1371/journal.pmed.1002716)
Supplement: S5 Table — NCD, noncommunicable disease; OLS, ordinary least squares. (DOCX) [file pmed.1002716.s007.docx]

|  |  | **Males** |  |  |  | **Females** |  |  |  |
| --- | --- | --- | --- | --- | --- | --- | --- | --- | --- |
|  |  | **6 diseases** |  | **13 diseases** |  | **6 diseases** |  | **13 diseases** |  |
| **Variable** |  | **Base model, scaled** | **+ disease interactions^‡^** | **Base model, scaled** | **+ disease interactions^‡^** | **Base model, scaled** | **+ disease interactions^‡^** | **Base model, scaled** | **+ disease interactions^‡^** |
| DISEASE-PHASE MAIN EFFECTS | | | | | | | | | |
| Disease main effects – first year of diagnosis | | | | | | | | | |
| Cancer |  | 7065 | 7529 |  |  | 9307 | 9396 |  |  |
|  | Lung |  |  | 7624 | 8207 |  |  | 8160 | 8174 |
|  | Colorectal |  |  | 13686 | 14759 |  |  | 13713 | 14036 |
|  | Breast |  |  |  |  |  |  | 6943 | 7045 |
|  | Prostate |  |  | 3042 | 3222 |  |  |  |  |
|  | Other |  |  | 8193 | 8728 |  |  | 8539 | 8587 |
| CVD |  | 7137 | 7542 |  |  | 5725 | 5676 |  |  |
|  | IHD |  |  | 6064 | 6467 |  |  | 4682 | 4701 |
|  | Stroke |  |  | 4520 | 4791 |  |  | 4992 | 5014 |
|  | Other CVD |  |  | 9433 | 10159 |  |  | 7111 | 7265 |
| DM |  | 922 | 562 | 898 | 555 | 598 | 291 | 597 | 302 |
| Chronic LLK |  | 7470 | 7223 |  |  | 7067 | 6438 |  |  |
|  | Chronic lung |  |  | 6484 | 6490 |  |  | 6013 | 5658 |
|  | CKD |  |  | 8408 | 8597 |  |  | 8306 | 8001 |
|  | CLD |  |  | 8188 | 8341 |  |  | 7676 | 7383 |
| Neurological |  | 5864 | 5340 | 5607 | 5101 | 4124 | 3652 | 4012 | 3551 |
| Musculoskeletal |  | 4796 | 4816 | 4726 | 4767 | 5709 | 5660 | 5647 | 5608 |
| Disease main effects – last year of life if dying of disease | | | | | | | | | |
| Cancer |  | 5734 | 5664 |  |  | 6475 | 6054 |  |  |
|  | Lung |  |  | 3800 | 3823 |  |  | 4647 | 4268 |
|  | Colorectal |  |  | 6598 | 6811 |  |  | 6994 | 6720 |
|  | Breast |  |  |  |  |  |  | 5713 | 5342 |
|  | Prostate |  |  | 4026 | 3942 |  |  |  |  |
|  | Other |  |  | 6602 | 6758 |  |  | 7043 | 6712 |
| CVD |  | 5316 | 5339 |  |  | 5644 | 5330 |  |  |
|  | IHD |  |  | 3329 | 3368 |  |  | 4115 | 3922 |
|  | Stroke |  |  | 2624 | 2694 |  |  | 3480 | 3398 |
|  | Other CVD |  |  | 8156 | 8723 |  |  | 7736 | 7860 |
| DM |  | 5983 | 5224 | 5679 | 4955 | 5968 | 4950 | 5531 | 4653 |
| Chronic LLK |  | 5175 | 4749 |  |  | 4936 | 4123 |  |  |
|  | Chronic lung |  |  | 4642 | 4589 |  |  | 4440 | 3936 |
|  | CKD |  |  | 12882 | 13034 |  |  | 15096 | 14895 |
|  | CLD |  |  | 4836 | 4563 |  |  | 7565 | 7269 |
| Neurological |  | 2545 | 1991 | 2632 | 2121 | 1936 | 1481 | 2008 | 1570 |
| Musculoskeletal |  | 11710 | 12023 | 11675 | 12017 | 8915 | 8784 | 8693 | 8599 |
| Disease main effects – prevalent years of diagnosis | | | | | | | | | |
| Cancer |  | 1384 | 1305 |  |  | 1288 | 1059 |  |  |
|  | Lung |  |  | 2026 | 2060 |  |  | 2342 | 2066 |
|  | Colorectal |  |  | 1645 | 1649 |  |  | 1259 | 1082 |
|  | Breast |  |  |  |  |  |  | 576 | 417 |
|  | Prostate |  |  | 671 | 624 |  |  |  |  |
|  | Other |  |  | 1882 | 1892 |  |  | 1558 | 1372 |
| CVD |  | 1514 | 1346 |  |  | 1399 | 1092 |  |  |
|  | IHD |  |  | 919 | 873 |  |  | 995 | 842 |
|  | Stroke |  |  | 572 | 492 |  |  | 547 | 370 |
|  | Other CVD |  |  | 1365 | 1394 |  |  | 1319 | 1241 |
| DM |  | 1007 | 500 | 989 | 507 | 948 | 504 | 936 | 513 |
| Chronic LLK |  | 2491 | 1672 |  |  | 2368 | 1433 |  |  |
|  | Chronic lung |  |  | 2090 | 1619 |  |  | 2047 | 1412 |
|  | CKD |  |  | 3335 | 3025 |  |  | 3288 | 2763 |
|  | CLD |  |  | 1344 | 844 |  |  | 1153 | 632 |
| Neurological |  | 1397 | 466 | 1372 | 513 | 1000 | 375 | 987 | 396 |
| Musculoskeletal |  | 1115 | 754 | 1064 | 770 | 1283 | 1001 | 1249 | 1006 |
| DISEASE COMORBIDITY INTERACTIONS | | | | | | | | | |
| Cancer & CVD |  |  | -163 |  | -243 |  | -166 |  | -275 |
| Cancer & DM |  |  | -166 |  | -192 |  | 29 |  | 67 |
| Cancer & LLK |  |  | -22 |  | -358 |  | -236 |  | -380 |
| Cancer and Neuro |  |  | 1249 |  | 975 |  | 1171 |  | 1078 |
| Cancer and MS |  |  | 205 |  | 269 |  | 194 |  | 228 |
| CVD and DM |  |  | 519 |  | 466 |  | 549 |  | 473 |
| CVD and LLK |  |  | 463 |  | 162 |  | 707 |  | 443 |
| CVD and Neuro |  |  | 322 |  | 280 |  | 384 |  | 317 |
| CVD and MS |  |  | 375 |  | 166 |  | 243 |  | 95 |
| DM and LLK |  |  | 410 |  | 267 |  | 473 |  | 332 |
| DM and Neuro |  |  | 1222 |  | 1224 |  | 782 |  | 784 |
| DM and MS |  |  | 233 |  | 248 |  | 111 |  | 126 |
| LLK and Neuro |  |  | 665 |  | 567 |  | 839 |  | 676 |
| LLK and MS |  |  | 810 |  | 631 |  | 490 |  | 348 |
| Neuro and MS |  |  | 723 |  | 740 |  | 581 |  | 597 |

‡ The disease–disease interaction models all included 15 dummy variables for each pairwise combination of the six aggregated diseases: cancer, CVD, DM, LLK, Neuro and MS. Age and age-squared interactions with disease comorbidity interactions were also included.
