# Supplementary material for: Health system costs for individual and comorbid noncommunicable diseases: An analysis of publicly funded health events from New Zealand
Source: PLoS Med. 2019 Jan 8;16(1):e1002716. doi: 10.1371/journal.pmed.1002716 (PMC6324792; doi:10.1371/journal.pmed.1002716)
Supplement: S6 Table — GEE, general estimating equation; OLS, ordinary least squares; s.e., standard error. (DOCX) [file pmed.1002716.s008.docx]

**S6 Table.** Comparison of coefficients (s.e. in parentheses; $NZ for 2011) for three modelling approaches, for 6-diseases without comorbidity interactions: OLS regression on aggregated data (default method; as shown in Table 3); OLS regression on individual-level data; and OLS regression on individual-level data with GEE.

|  | **Males** |  |  | **Females** |  |  |
| --- | --- | --- | --- | --- | --- | --- |
| **Variable** | **OLS Aggregate data** | **OLS individual-level data** | **OLS individual-level data GEE** | **OLS Aggregate data** | **OLS individual-level data** | **OLS individual-level data GEE** |
| Intercept | 475 (37) | 475 (8) | 475 (9) | 601 (31) | 601 (6) | 601 (7) |
| Age | 215 (17) | 215 (4) | 215 (5) | 229 (12) | 229 (2) | 229 (3) |
| Age squared | 67 (6) | 67 (1) | 67 (1) | 103 (5) | 103 (1) | 103 (1) |
| Disease main effects – first year of diagnosis |  |  |  |  |  |  |
| Cancer | 13007 (207) | 13007 (43) | 13007 (121) | 16309 (193) | 16309 (39) | 16309 (125) |
| CVD | 12582 (160) | 12582 (34) | 12582 (91) | 10002 (164) | 10002 (33) | 10002 (107) |
| DM | 741 (191) | 741 (40) | 741 (51) | 436 (167) | 436 (34) | 436 (34) |
| Chronic LLK | 12017 (255) | 12017 (53) | 12017 (168) | 11134 (237) | 11134 (48) | 11134 (168) |
| Neurological | 8551 (136) | 8551 (28) | 8551 (84) | 5004 (96) | 5004 (20) | 5004 (46) |
| Musculoskeletal | 5480 (129) | 5480 (27) | 5480 (55) | 7776 (127) | 7776 (26) | 7776 (62) |
| Disease main effects – last year of life if dying of disease |  |  |  |  |  |  |
| Cancer | 15380 (316) | 15380 (66) | 15380 (195) | 16422 (285) | 16422 (58) | 16422 (201) |
| CVD | 13573 (486) | 13573 (102) | 13573 (493) | 14766 (522) | 14766 (107) | 14766 (409) |
| DM | 23848 (1053) | 23848 (221) | 23848 (2047) | 26842 (1089) | 26842 (222) | 26842 (2803) |
| Chronic LLK | 15188 (814) | 15188 (171) | 15188 (757) | 15481 (735) | 15481 (150) | 15481 (869) |
| Neurological | 6401 (953) | 6401 (200) | 6401 (403) | 5539 (825) | 5539 (169) | 5539 (335) |
| Musculoskeletal | 26896 (3097) | 26896 (649) | 26896 (2902) | 21449 (1902) | 21449 (389) | 21449 (1560) |
| Disease main effects – prevalent years |  |  |  |  |  |  |
| Cancer | 2861 (87) | 2861 (18) | 2861 (43) | 2872 (71) | 2872 (14) | 2872 (40) |
| CVD | 2428 (58) | 2428 (12) | 2428 (28) | 2407 (59) | 2407 (12) | 2407 (36) |
| DM | 1877 (63) | 1877 (13) | 1877 (38) | 1704 (55) | 1704 (11) | 1704 (35) |
| Chronic LLK | 4914 (102) | 4914 (21) | 4914 (104) | 4251 (86) | 4251 (18) | 4251 (87) |
| Neurological | 2224 (59) | 2224 (12) | 2224 (33) | 1439 (40) | 1439 (8) | 1439 (18) |
| Musculoskeletal | 1265 (45) | 1265 (10) | 1265 (18) | 1814 (45) | 1814 (9) | 1814 (22) |
| Disease age interactions |  |  |  |  |  |  |
| Cancer – first year of diagnosis | -1836 (129) | -1836 (27) | -1836 (81) | -1378 (98) | -1378 (20) | -1378 (61) |
| Cancer – last year of life if dying of disease | -4543 (222) | -4543 (46) | -4543 (194) | -4411 (172) | -4411 (35) | -4411 (142) |
| Cancer – prevalent years | -448 (51) | -448 (11) | -448 (28) | -629 (39) | -629 (8) | -629 (21) |
| CVD – first year of diagnosis | -633 (90) | -633 (19) | -633 (52) | -922 (85) | -922 (17) | -922 (56) |
| CVD – last year of life if dying of disease | -3614 (381) | -3614 (80) | -3614 (698) | -4497 (394) | -4497 (81) | -4497 (354) |
| CVD – prevalent years | -27 (34) | -27 (7) | -27 (19) | -159 (31) | -159 (6) | -159 (22) |
| DM – first year of diagnosis | 199 (137) | 199 (29) | 199 (38) | -64 (108) | -64 (22) | -64 (25) |
| DM – last year of life if dying of disease | -11502 (979) | -11502 (205) | -11502 (1983) | -15450 (969) | -15450 (198) | -15450 (5218) |
| DM – prevalent years | -143 (37) | -143 (8) | -143 (19) | -134 (29) | -134 (6) | -134 (13) |
| Chronic LLK– first year of diagnosis | 50 (120) | 50 (25) | 50 (76) | 12 (100) | 12 (20) | 12 (54) |
| Chronic LLK– last year of life if dying of disease | -6017 (705) | -6017 (148) | -6017 (1631) | -6652 (614) | -6652 (125) | -6652 (1632) |
| Chronic LLK – prevalent years | -451 (48) | -451 (10) | -451 (42) | -104 (36) | -104 (7) | -104 (23) |
| Neurological – first year of diagnosis | 822 (59) | 822 (12) | 822 (27) | 789 (39) | 789 (8) | 789 (14) |
| Neurological – last year of life if dying of disease | -1450 (553) | -1450 (116) | -1450 (233) | -1636 (568) | -1636 (116) | -1636 (244) |
| Neurological – prevalent years | 96 (30) | 96 (6) | 96 (12) | 72 (19) | 72 (4) | 72 (6) |
| Musculoskeletal – first year of diagnosis | 1174 (72) | 1174 (15) | 1174 (29) | 615 (57) | 615 (12) | 615 (23) |
| Musculoskeletal – last year of life if dying of disease | -3655 (1788) | -3655 (375) | -3655 (1490) | -4705 (1101) | -4705 (225) | -4705 (1015) |
| Musculoskeletal – prevalent years | 274 (26) | 274 (5) | 274 (9) | 120 (20) | 120 (4) | 120 (7) |
| Disease age-squared interactions |  |  |  |  |  |  |
| Cancer – first year of diagnosis | 209 (72) | 209 (15) | 209 (44) | -342 (56) | -342 (11) | -342 (37) |
| Cancer – last year of life if dying of disease | 61 (108) | 61 (23) | 61 (85) | -192 (85) | -192 (17) | -192 (65) |
| Cancer – prevalent years | -39 (28) | -39 (6) | -39 (14) | -28 (22) | -28 (4) | -28 (11) |
| CVD – first year of diagnosis | -356 (52) | -356 (11) | -356 (28) | -147 (46) | -147 (9) | -147 (28) |
| CVD – last year of life if dying of disease | -37 (148) | -37 (31) | -37 (236) | 67 (126) | 67 (26) | 67 (93) |
| CVD – prevalent years | -40 (19) | -40 (4) | -40 (10) | -63 (16) | -63 (3) | -63 (10) |
| DM – first year of diagnosis | 160 (70) | 160 (15) | 160 (19) | 233 (50) | 233 (10) | 233 (11) |
| DM – last year of life if dying of disease | 1105 (444) | 1105 (93) | 1105 (665) | 2210 (369) | 2210 (75) | 2210 (1756) |
| DM – prevalent years | -60 (23) | -60 (5) | -60 (12) | -58 (16) | -58 (3) | -58 (8) |
| Chronic LLK– first year of diagnosis | -335 (67) | -335 (14) | -335 (44) | -410 (52) | -410 (11) | -410 (29) |
| Chronic LLK– last year of life if dying of disease | 752 (280) | 752 (59) | 752 (606) | 730 (240) | 730 (49) | 730 (533) |
| Chronic LLK – prevalent years | -203 (27) | -203 (6) | -203 (24) | -296 (18) | -296 (4) | -296 (14) |
| Neurological – first year of diagnosis | -393 (30) | -393 (6) | -393 (14) | -96 (18) | -96 (4) | -96 (7) |
| Neurological – last year of life if dying of disease | -162 (218) | -162 (46) | -162 (82) | -126 (176) | -126 (36) | -126 (63) |
| Neurological – prevalent years | -110 (14) | -110 (3) | -110 (6) | -44 (9) | -44 (2) | -44 (3) |
| Musculoskeletal – first year of diagnosis | 17 (34) | 17 (7) | 17 (13) | -193 (29) | -193 (6) | -193 (12) |
| Musculoskeletal – last year of life if dying of disease | -1618 (812) | -1618 (170) | -1618 (647) | -661 (444) | -661 (91) | -661 (318) |
| Musculoskeletal – prevalent years | 7 (12) | 7 (3) | 7 (4) | -60 (10) | -60 (2) | -60 (4) |
