# Supplementary material for: Health system costs for individual and comorbid noncommunicable diseases: An analysis of publicly funded health events from New Zealand
Source: PLoS Med. 2019 Jan 8;16(1):e1002716. doi: 10.1371/journal.pmed.1002716 (PMC6324792; doi:10.1371/journal.pmed.1002716)
Supplement: S7 Table — OLS, ordinary least squares; s.e., standard error. (DOCX) [file pmed.1002716.s009.docx]

**S7 Table**. Comparison of coefficients (s.e. in parentheses; $NZ for 2011) for OLS regression on individual level data (as shown in S6 Table) compared to fixed effects regression on individual-level data utilizing within person changes by year in disease status and health expenditure.

|  | **Males** |  | **Females** |  |
| --- | --- | --- | --- | --- |
| **Variable** | **OLS** | **Fixed effects** | **OLS** | **Fixed effects** |
| Intercept |  |  |  |  |
| Age | 215 (4) | 154 (15) | 229 (2) | 71 (19) |
| Age squared | 67 (1) | 86 (2) | 103 (1) | 67 (3) |
| Disease main effects – first year of diagnosis |  |  |  |  |
| Cancer | 13007 (43) | 16129 (39) | 16309 (39) | 13024 (44) |
| CVD | 12582 (34) | 9397 (34) | 10002 (33) | 12302 (34) |
| DM | 741 (40) | 322 (35) | 436 (34) | 737 (42) |
| Chronic LLK | 12017 (53) | 9537 (49) | 11134 (48) | 10481 (55) |
| Neurological | 8551 (28) | 4650 (20) | 5004 (20) | 8076 (29) |
| Musculoskeletal | 5480 (27) | 7197 (26) | 7776 (26) | 5196 (28) |
| Disease main effects – last year of life if dying of disease |  |  |  |  |
| Cancer | 15380 (66) | 12456 (64) | 16422 (58) | 11880 (74) |
| CVD | 13573 (102) | 8024 (118) | 14766 (107) | 6498 (114) |
| DM | 23848 (221) | -15796 (245) | 26842 (222) | -12406 (245) |
| Chronic LLK | 15188 (171) | 4924 (166) | 15481 (150) | 3933 (191) |
| Neurological | 6401 (200) | 2340 (183) | 5539 (169) | 4036 (216) |
| Musculoskeletal | 26896 (649) | 9766 (415) | 21449 (389) | 4626 (718) |
| Disease main effects – prevalent years |  |  |  |  |
| Cancer | 2861 (18) | 5520 (29) | 2872 (14) | 5006 (35) |
| CVD | 2428 (12) | 2314 (28) | 2407 (12) | 2499 (29) |
| DM | 1877 (13) | 487 (28) | 1704 (11) | 724 (34) |
| Chronic LLK | 4914 (21) | 2737 (42) | 4251 (18) | 3129 (49) |
| Neurological | 2224 (12) | 1220 (17) | 1439 (8) | 1991 (26) |
| Musculoskeletal | 1265 (10) | 1421 (22) | 1814 (9) | 1118 (24) |
| Disease age interactions |  |  |  |  |
| Cancer – first year of diagnosis | -1836 (27) | -1244 (20) | -1378 (20) | -1606 (28) |
| Cancer – last year of life if dying of disease | -4543 (46) | -2617 (39) | -4411 (35) | -2873 (54) |
| Cancer – prevalent years | -448 (11) | -1117 (15) | -629 (8) | -553 (21) |
| CVD – first year of diagnosis | -633 (19) | -788 (18) | -922 (17) | -504 (19) |
| CVD – last year of life if dying of disease | -3614 (80) | -2922 (89) | -4497 (81) | -512 (91) |
| CVD – prevalent years | -27 (7) | -109 (16) | -159 (6) | 98 (17) |
| DM – first year of diagnosis | 199 (29) | 60 (22) | -64 (22) | 307 (29) |
| DM – last year of life if dying of disease | -11502 (205) | 6284 (223) | -15450 (198) | 5950 (232) |
| DM – prevalent years | -143 (8) | 51 (15) | -134 (6) | 150 (20) |
| Chronic LLK– first year of diagnosis | 50 (25) | 57 (21) | 12 (20) | 142 (26) |
| Chronic LLK– last year of life if dying of disease | -6017 (148) | -50 (141) | -6652 (125) | 741 (168) |
| Chronic LLK – prevalent years | -451 (10) | -15 (18) | -104 (7) | -260 (24) |
| Neurological – first year of diagnosis | 822 (12) | 772 (8) | 789 (8) | 814 (12) |
| Neurological – last year of life if dying of disease | -1450 (116) | 63 (128) | -1636 (116) | -180 (132) |
| Neurological – prevalent years | 96 (6) | 100 (7) | 72 (4) | 186 (12) |
| Musculoskeletal – first year of diagnosis | 1174 (15) | 654 (11) | 615 (12) | 1181 (15) |
| Musculoskeletal – last year of life if dying of disease | -3655 (375) | -1079 (246) | -4705 (225) | 9403 (453) |
| Musculoskeletal – prevalent years | 274 (5) | 110 (10) | 120 (4) | 306 (13) |
| Disease age-squared interactions |  |  |  |  |
| Cancer – first year of diagnosis | 209 (15) | -300 (11) | -342 (11) | 159 (15) |
| Cancer – last year of life if dying of disease | 61 (23) | -289 (19) | -192 (17) | -104 (26) |
| Cancer – prevalent years | -39 (6) | -96 (8) | -28 (4) | -71 (11) |
| CVD – first year of diagnosis | -356 (11) | -97 (9) | -147 (9) | -317 (11) |
| CVD – last year of life if dying of disease | -37 (31) | 330 (28) | 67 (26) | -198 (35) |
| CVD – prevalent years | -40 (4) | 19 (7) | -63 (3) | 40 (8) |
| DM – first year of diagnosis | 160 (15) | 246 (10) | 233 (10) | 143 (14) |
| DM – last year of life if dying of disease | 1105 (93) | -73 (84) | 2210 (75) | -456 (104) |
| DM – prevalent years | -60 (5) | 84 (7) | -58 (3) | 57 (10) |
| Chronic LLK– first year of diagnosis | -335 (14) | -262 (11) | -410 (11) | -185 (14) |
| Chronic LLK– last year of life if dying of disease | 752 (59) | -536 (54) | 730 (49) | -495 (66) |
| Chronic LLK – prevalent years | -203 (6) | -49 (8) | -296 (4) | 14 (12) |
| Neurological – first year of diagnosis | -393 (6) | -78 (3) | -96 (4) | -374 (6) |
| Neurological – last year of life if dying of disease | -162 (46) | -237 (39) | -126 (36) | -238 (50) |
| Neurological – prevalent years | -110 (3) | -34 (3) | -44 (2) | -109 (5) |
| Musculoskeletal – first year of diagnosis | 17 (7) | -173 (6) | -193 (6) | 17 (7) |
| Musculoskeletal – last year of life if dying of disease | -1618 (170) | -541 (100) | -661 (91) | -3980 (202) |
| Musculoskeletal – prevalent years | 7 (3) | -26 (4) | -60 (2) | 23 (5) |

Coefficients are in $NZ 2011.
