# Supplementary figures and images for: Health system costs for individual and comorbid noncommunicable diseases: An analysis of publicly funded health events from New Zealand
Source: PLoS Med. 2019 Jan 8;16(1):e1002716. doi: 10.1371/journal.pmed.1002716 (PMC6324792; doi:10.1371/journal.pmed.1002716)

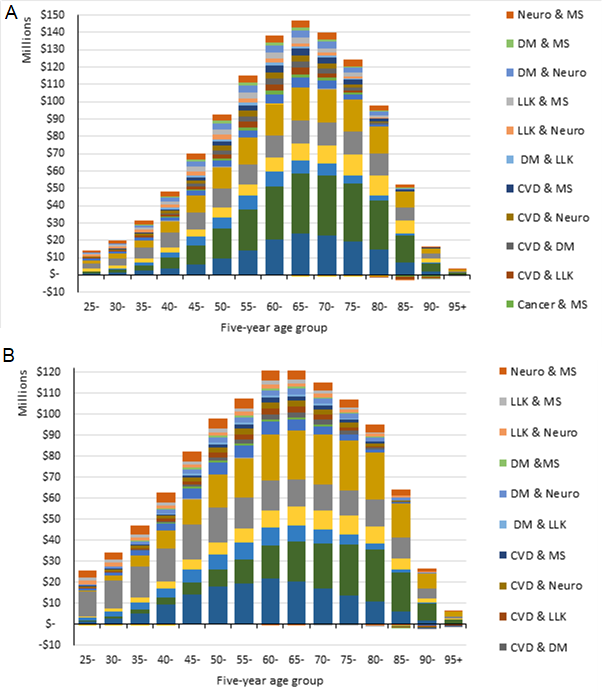

Supplement: S1 Fig — (TIF) [file pmed.1002716.s010.tif]

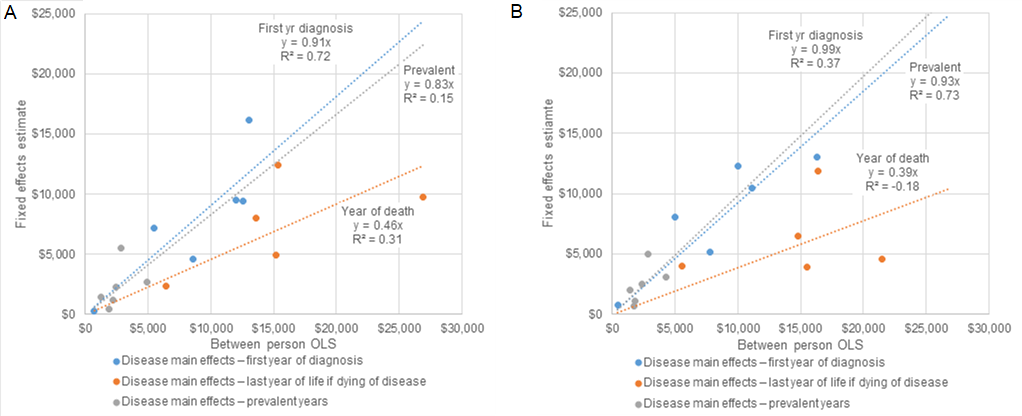

Supplement: S2 Fig — Coefficients are in NZ$ 2011. OLS, ordinary least squares. (TIF) [file pmed.1002716.s011.tif]
